# Conjugation between 3D and 2D aromaticity: does it really exist? The case of carborane-fused heterocycles†‡

**DOI:** 10.1039/d2sc03511a

**Published:** 2022-09-06

**Authors:** Dániel Buzsáki, Máté Barnabás Kovács, Evelyn Hümpfner, Zsófia Harcsa-Pintér, Zsolt Kelemen

**Affiliations:** MTA-BME Computation Driven Chemistry Group Műegyetem rkp 3. H-1111 Budapest Hungary; Department of Inorganic and Analytical Chemistry, Budapest University of Technology and Economics Műegyetem rkp 3. H-1111 Budapest Hungary kelemen.zsolt@vbk.bme.hu

## Abstract

Although several synthesized icosahedral carborane fused 2D π-ring systems are known, and even considerable conjugation has been noted between them in some cases, the phenomenon itself is not fully understood. Based on the results of our computational study, it can be concluded that the 2D aromatic character of the fused (*exo*) five-membered ring is low, even in cases where significant conjugation was proposed in previous studies. Moreover, the carborane moiety constricts the bonding properties of the *exo* ring, thus prohibiting or promoting different Lewis resonance structures. These results will shed further light on the design and electronic modulation of new carborane-based materials.

## Abstract 2

We have demonstrated that carborane fused heterocycles can definitely be considered as non-2D aromatic compounds; therefore, their aromatic character was significantly overestimated in earlier studies.

## Graphical abstract

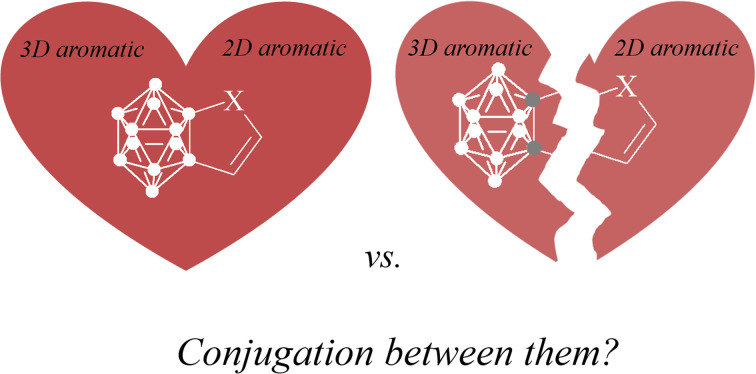

## Introduction

**Scheme 1:**
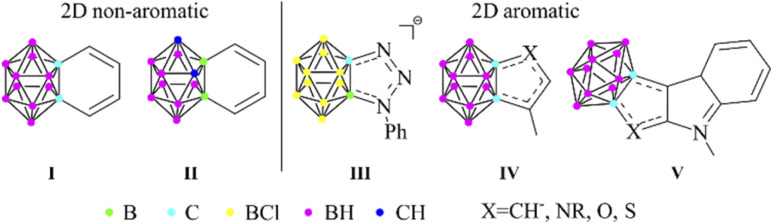
Carborane fused 2D ring systems.

Icosahedral *closo*-dicarbadodecaboranes (C_2_B_10_H_12_) are the most studied carboranes, since their possible application covers a wide range.^1^ These 3D cluster compounds show similarities to the organic aromatic molecules,^2^ as they are highly stable and can take part in electrophilic substitution reactions.^3^ The 26 skeletal electrons fill 13 orbitals *via* multi-center (mainly 3c2e) bonding, thus resulting in a fully delocalized system, often referred to as 3D aromaticity.^4^ In the last few years, the similarities and differences of 3D aromatic systems to their π-aromatic analogues have been highlighted in several pioneering studies.^5^ Moreover, the existence of planar carborane systems with combined σ- and π-aromatic ring currents was predicted^6^ and they were synthesized,^7^ further strengthening the close relationship between the two types of aromaticity. If the statement of Teixidor and Solà is true, and π- and 3D aromaticity are two sides of the same coin,^5*b*^ then the question arises whether noticeable conjugation can be achieved between them. Although significant interaction was found in some arylated *closo*-CB_11_H_12_^−^ anions,^8^ it could possibly be augmented by fusing the two compounds into 3D–2D fused systems. Benzocarborane I (Scheme 1) was the first example of this group;^9*a*^ however, these compounds (I and II) have localized double bonds,^9^ thus no aromatic character in the *exo* ring could be detected.

On the other hand, Lavallo demonstrated the aromatic character of the fused ring in the negatively charged HCB_11_Cl_11_^−^ based system (III).^10^ Furthermore, a recent study on heterocycles fused with *ortho*-carborane revealed a noticeable change in the aromatic character of the *exo* ring connected *via* its π-donor atoms, which was proven by NMR spectroscopy and computational studies (IV).^11^ It was suggested that the negative hyperconjugation is responsible for this phenomenon; however, no further explanation was given on the increased aromaticity of the fused ring. Moreover, the interaction of the fusion between carboranes and chalcogen-containing compounds (V) was also interpreted as 3D–2D conjugation.^12^ Very recently, the computational study of Teixidor and Solà has confirmed the earlier experiences regarding the non-aromatic character and the lack of conjugation in the benzocarborane derivatives;^13^ however, the previously reported conjugating ability of five-membered ring systems was not discussed.

## Results and discussion

Due to the discrepancy between the previous results, the phenomenon itself is still a matter of debate; the effect of the ring size and the positions of different heteroatoms is not precisely clear.^9–13^ Why does the size of the ring have such a great influence on the interaction between the systems? Can the effect be explained with the negative hyperconjugation in all cases? Fully understanding the determining effects of this interaction would be highly desirable for both fundamental and practical aspects and would allow us to design and modulate new materials for a given application.

**Fig. 1:**
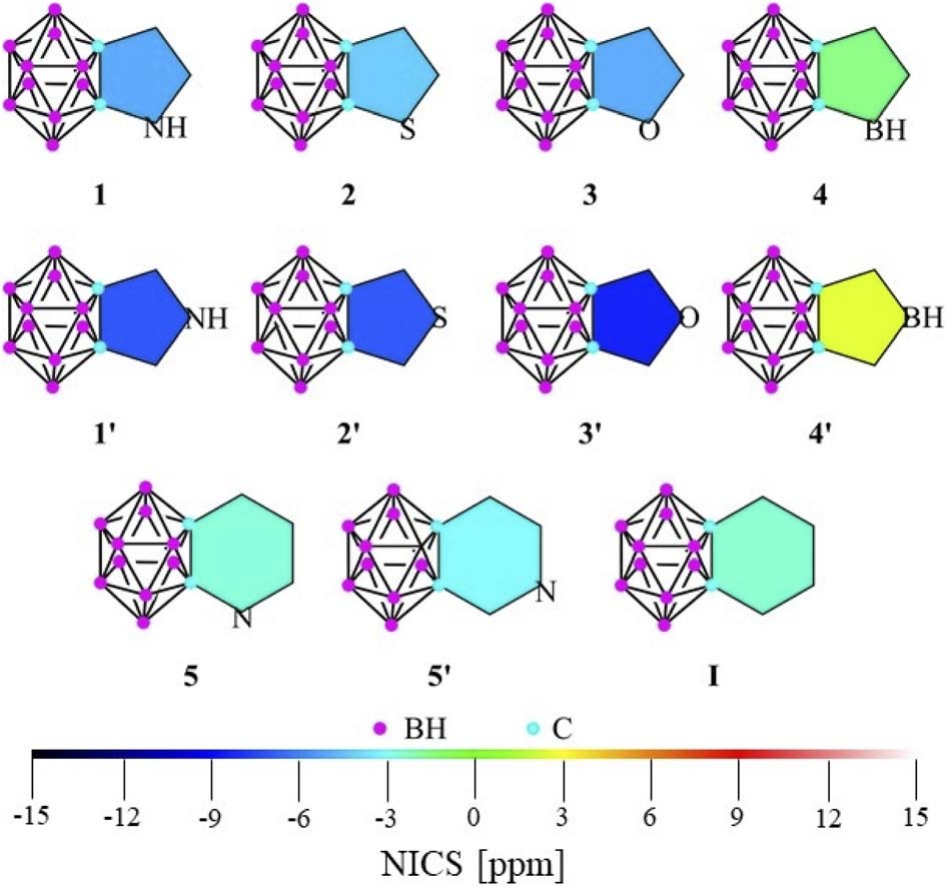
The calculated NICS(1) values of the investigated systems at the B3LYP/cc-pVTZ//B3LYP/6-311+G** level of theory.

While being not directly measurable, several sub-criteria can contribute to the overall definition of aromaticity.^14^ Among the various theoretical approaches (computational details in the ESI‡), calculations on the nucleus independent chemical shift (NICS) are the most widely applied descriptors of aromaticity. Although Xie studied several systems fused *via* the C_c_–C_c_ (carbon–carbon bond of the cluster) bond of the *ortho*-carborane (IV, Scheme 1),^11^ we extended the set of possibly applicable heterocycles with different aromatic characters to perform a widespread comparison (Fig. 1). In this work, we use NICS(1) and NICS(0) values in order to contrast our results with a previous study.^11^ However, it should be mentioned that according to Schleyer, NICS(1)_zz_ has been found to provide the most accurate results^15^ and showed a similar trend for our investigated systems (see Table S1 in the ESI‡).

While focusing on five-membered rings, NICS(1) indices (Fig. 1, tabulated data and NICS(0) values can be found in Table S1‡) exhibit higher aromatic character in the *C*_2v_ symmetric 1′–3′ structures (by 3–4 ppm) than their respective isomers with *C*_s_ symmetry (1–3). As no electronegative heteroatom is connected directly to the cluster in 1′–3′, the negative hyperconjugation effect^11^ cannot solely explain the increased aromaticity. However, in 4 and 4′, the anti-aromatic character is increased in the symmetric system, as the borole moiety is highly anti-aromatic in itself (see NICS(1) data in Table S1 in the ESI‡). In the case of the fused systems with six-membered rings, the difference between the NICS(1) values of the two isomers (5, 5′) becomes negligible and very similar to the value of benzocarborane (I). Furthermore, these indices generally show lower aromaticity compared to their five-membered counterparts, which cannot be explained simply by the well-known effect of the ring size,^14*a*^ since the results of these calculations for the parent heterocycles are similar to each other (Table S1‡).

**Scheme 2:**
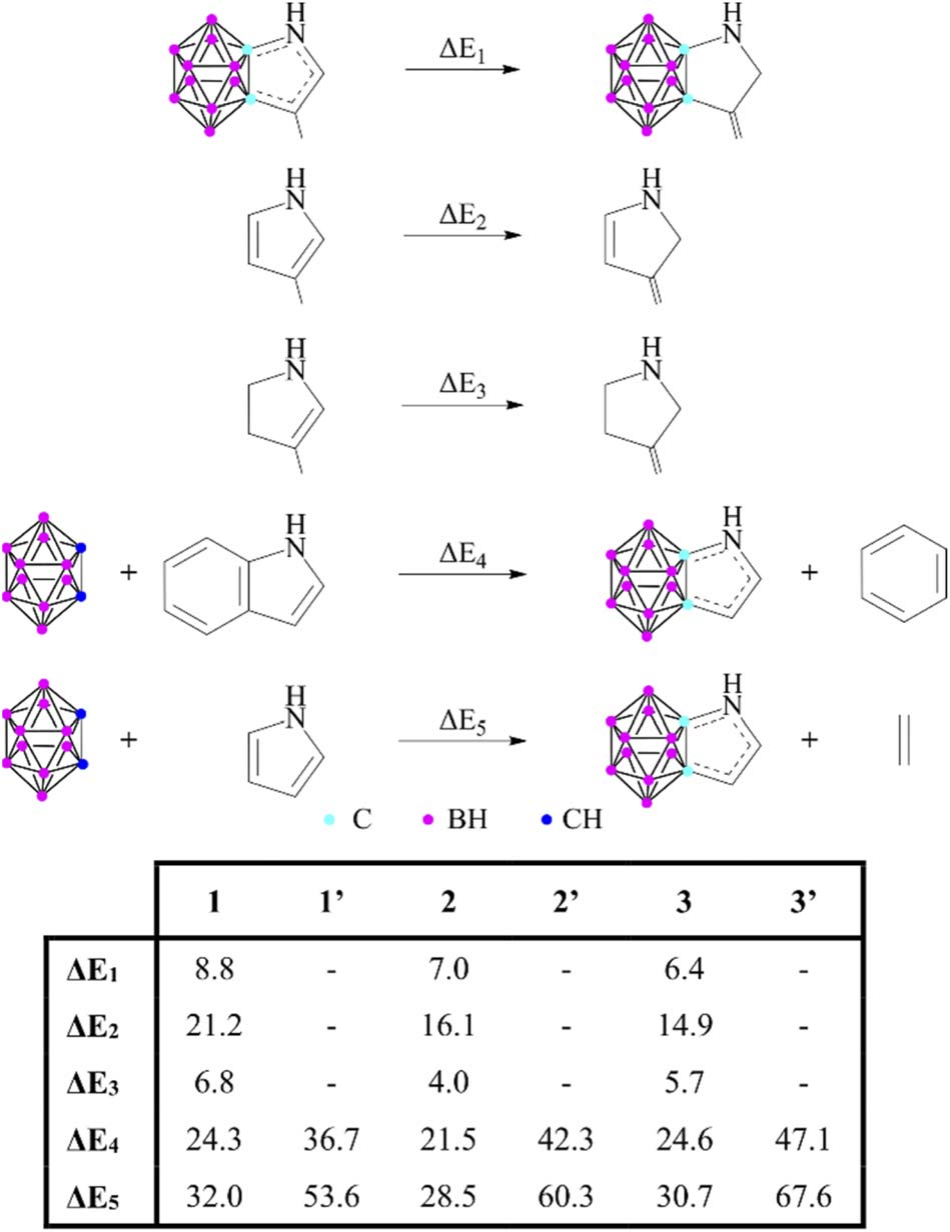
Various reactions to estimate the aromatic stabilization of 1–3 and 1′–3′. Reaction energies (in kcal mol^−1^) are calculated at the B3LYP/6-311+G** level of theory.

Some unanswered questions still remained; therefore, following the advice of Solà,^16^ we expanded the description of aromaticity to include a set of indicators that are based on different properties to either challenge or support the results. First, the thermodynamic stability of these systems *via* energy-based measures was investigated (Scheme 2). The isomerization stabilization energy (Δ*E*_1_) applied by Xie^11^ suggested lower aromatic character compared to the parent heterocycles (Δ*E*_2_). On the other hand, it should be noted that Δ*E*_1_ can also describe the conjugation between a sole double bond and the lone pair of the heteroatom; hence they should be compared to their partially saturated counterparts (Δ*E*_3_). As the values demonstrate, Δ*E*_1_ and Δ*E*_3_ are very similar to each other, which suggests a low degree of aromatic conjugation induced by the carborane moiety.

**Scheme 3:**
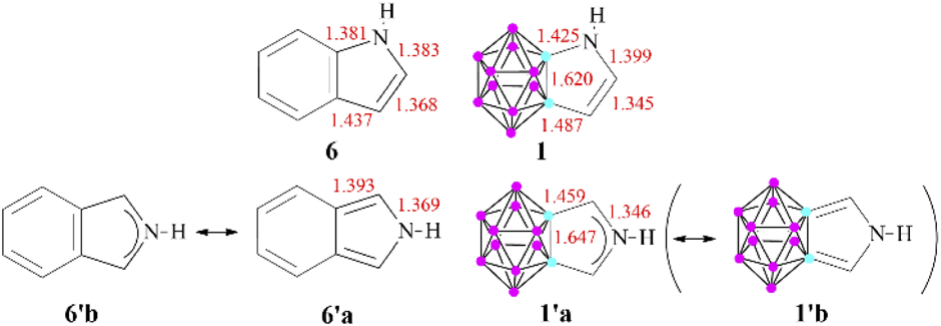
Selected bond lengths (in Å) for the pyrrole derivatives (B3LYP/6-311+G**). The elongated C_c_–C bond lengths in 1′ demonstrates= the low contribution of the 1′b resonance structure.

These results can be related to the reported ^1^H NMR data,^11^ as chemical shifts measured in the fused systems are more reminiscent of their corresponding α,β-unsaturated derivatives^17^ than the aromatic compounds (Table S2 in the ESI‡), showing that the delocalization within the *exo* ring has a definitive influence on the magnetic shielding compared to the effect of the carborane system. Furthermore, other isodesmic reactions (Δ*E*_4_ and Δ*E*_5_) also supported the low degree of aromatic conjugation as they were highly positive in all investigated cases; moreover, similarly to the NICS(1) values, they are strongly affected by the position of the heteroatoms. Highest isodesmic reaction energies could be found for symmetric systems, which also possess the strongest aromatic character according to the NICS(1) values. It is known that the more aromatic isoindole isomer (6′, Scheme 3) is less stable than indole (6) (by 7.5 kcal mol^−1^);^18^ however, even higher destabilization (by 21.6–39.9 kcal mol^−1^, see Table S6 in the ESI‡) occurs in the case of their carborane-fused counterparts.

To explain this destabilizing effect and to get more insight into the aromatic character of these compounds, the calculated bond lengths were compared to those of their purely organic oligo-aromatic counterparts (Scheme 3 shows the corresponding pyrrole derivatives; data for other compounds can be seen in the ESI in Fig. S1‡). In all investigated cases, the bond between the cluster and the neighboring *exo* ring atom can be described as a single bond, based on the bond length and also verified by the low ellipticity of electron density values in the bond critical points (see Fig. S2 in the ESI‡). For 1, a resonance structure similar to 6 can be envisaged; however, in the case of 1′, the primary Lewis structure of 6′^18^ (Table S3 in the ESI‡) cannot be applied, as the bond lengths of the C_c_–C (cluster-ring C–C) bonds are expanded (1.459 Å) and – in agreement with the high valency of the cluster carbon atoms – can only be considered as single bonds. This restriction prohibits the leading resonance structures containing the corresponding double bonds, thus inducing originally less weighted resonance structures to become more dominant. In fact, the bonding situation of the C–N–C unit possesses a stronger 3c4e bond character, which is also supported by the shorter C–N bonds and higher C–N Wiberg indices for 1′ compared to isoindole (1.31 and 1.22, respectively); therefore, delocalization occurs within the C–N–C system. According to the visualization of 1, 1′, and I in a GIMIC plot (Fig. S3 of the ESI‡), this phenomenon increases the density of the magnetic current in the center of the *exo* ring in 1′, thus affecting the calculated NICS(1) values in these systems.

**Fig. 2:**
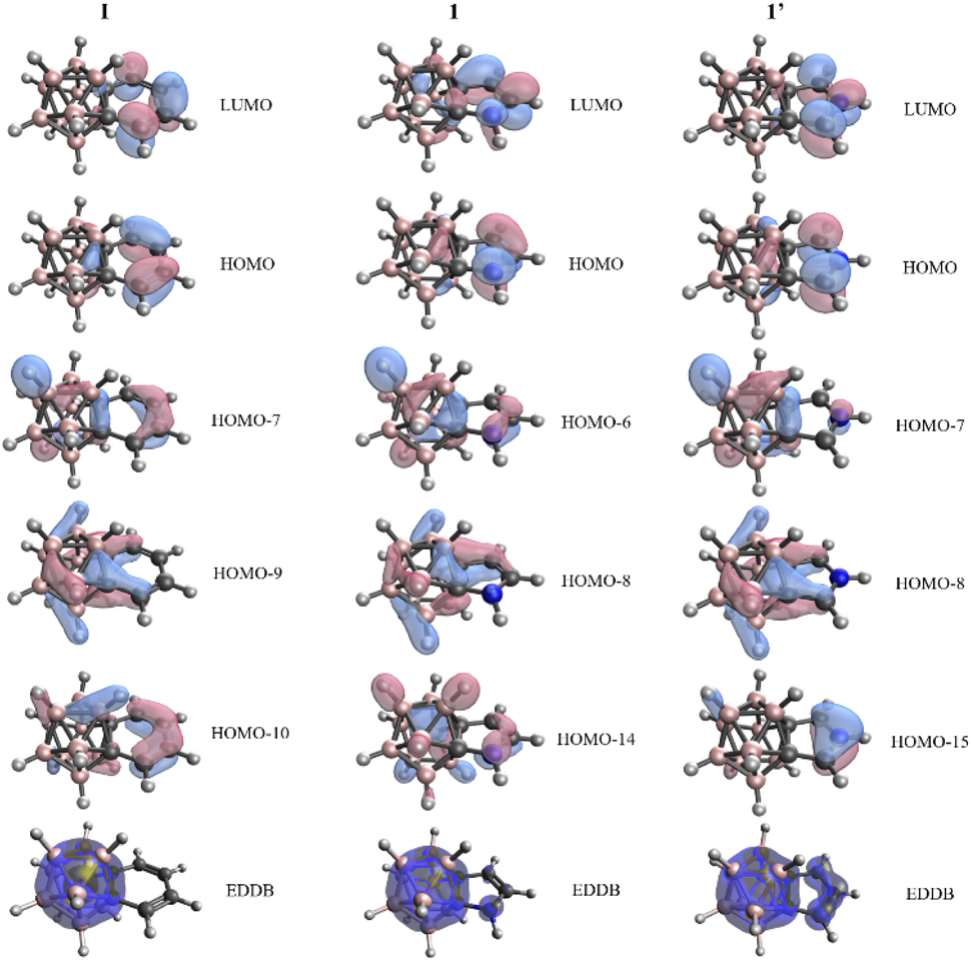
Kohn–Sham orbitals with significant π-character and the global electron density of delocalized bonds (EDDB) function in the cases of I, 1, and 1′. Calculations were performed at the B3LYP/cc-pVTZ//B3LYP/6-311+G** level of theory.

In view of these findings, the validity of the NICS values in the context of these systems is debatable, since all the other descriptors suggest non-aromatic character; thus significant 3D–2D conjugation for fused five-membered ring systems 1–3 and 1′–3′ cannot be observed. Similar conclusions could be obtained upon the investigation of the Kohn–Sham molecular orbitals (Fig. 2 and S4 in the ESI‡). Despite the existence of orbitals spreading over the fused rings and carborane moiety, the outer atoms of the *exo* system have more significant contribution to the π-orbitals, and an extensive variance does not occur among six- and five-membered ring systems. On top of this, we have performed calculations based on the global EDDB (electron density of delocalized bonds) function,^19^ which enables the representation of electrons delocalized over the whole system. The calculated data of I, 1, and 1′ (Fig. 2) show two separable delocalized systems in all cases; therefore, this method further supports our concept that the conjugation between 2D and 3D moieties is minor in all investigated systems.

As a last possible explanation, an obvious difference could be the ring size, which determines the distance of ghost atoms used for the NICS calculations from the carborane cluster. Our train of thought led us to the attempt of calculating the magnetic shielding of ghost atoms along and 1 Å away from the perpendicular bisector line of the C_c_–C_c_ bond drawn from the center of the *o*-carborane. Surprisingly, our results reveal that the magnetic field of the carborane has a noticeable impact in a huge radius around the cluster (Table S4 and Fig. S5 in the ESI‡) and is solely able to make a distinction between the NICS values of the five- and six-membered *exo* rings. Therefore, these calculations significantly overestimate the 2D aromatic character of the fused rings, especially for five-membered rings, and it can be established that the conjugation between the 3D and the *exo* system is minor and similar to the notoriously 2D non-aromatic benzocarborane. Our observation broadens the number of studies where the application of NICS failed.^20^ For example, in the hydrogen-bonded (HF)_3_ hexagon, a negative NICS value was obtained, but it resulted from local paratropic currents at each HF molecule and not a global diatropic ring current as was shown by Sundholm.^20*a*^ Similar to our work, a study by Frenking and co-workers confirmed the non-aromatic character of N_6_H_6_^2+^ and C_2_N_4_H_6_ systems based on electronic indices, although their reported NICS indices showed negative values.^20*b*^ On top of this, Bultinck and co-workers demonstrated that there is no one-to-one relationship between a NICS value and the magnetically induced current density.^20*c*^

In summary, these examples showed that NICS should be utilized carefully (despite being a very powerful tool), as these values only represent the integrated data of the induced ring currents without showing their accurate pattern, which can also be important while determining aromaticity.^16,21^

**Fig. 3:**
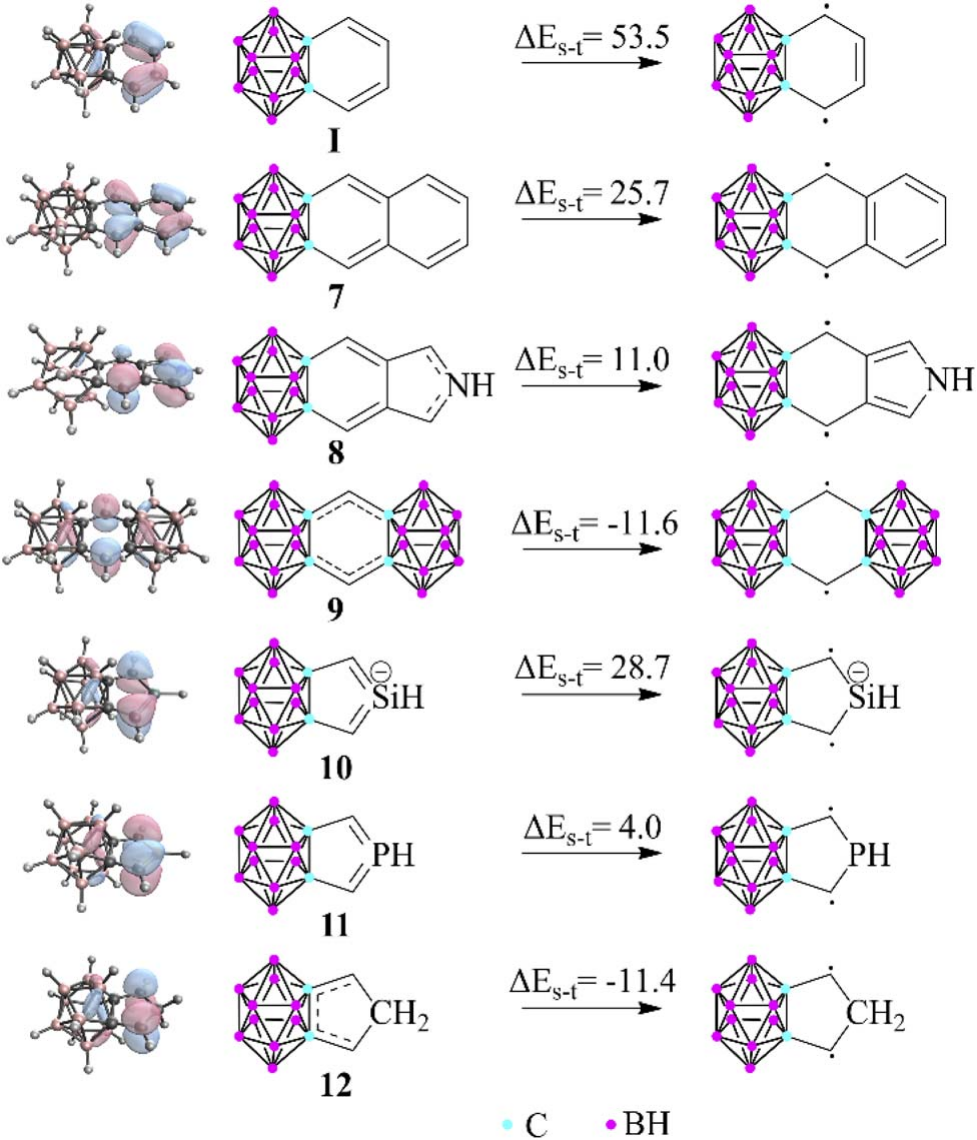
Singlet–triplet gap of selected fused compounds and the HOMO of the closed shell systems (B3LYP/6-311+G**, in kcal mol^−1^). The escalating localization of the HOMO clearly demonstrates the increased biradicaloid character. More data can be found in the ESI in Table S5 and Fig. S6, S7.‡

Apart from the investigation of the aromatic character of the fused ring, another interesting observation of this study is that the carborane lacks the ability to form double bonds with the outer member atoms of the *exo* rings, thus promoting generally less dominant resonance structures. To test the limitations of this phenomenon, we chose several unique structures (Fig. 3) in which the weights of the original principal resonance structures are altered; therefore, their geometry and aromatic properties can be ambiguous. Moreover, certain compounds were also considered in which the formation of all common Lewis structures is disabled; thus the system is forced to solve its electronic problems in inconvenient ways. First, the benzene-fused *exo* ring was extended to oligo-aromatic compounds (7, 8). It should be mentioned that the singlet–triplet gap decreases, and the HOMO orbitals are less polarized towards the outer ring, which suggests the increased weight of the biradicaloid character. By squeezing the *exo* ring between two clusters (9), the system is trying to prevent conjugation at any cost, pushing their electrons to the unfused C atoms, resulting in a more stable triplet state (by 11.6 kcal mol^−1^) compared to the closed-shell singlet system.

In the case of the symmetric phosphole and silolide anion derivatives (10 and 11), the carborane unit is able to planarize the phosphorus^22^ and silicon centers,^23^ in agreement with the 3c4e bond delocalization. Therefore, the effect is strong enough to compensate for the energy of the planarization, although it should be noted that the singlet–triplet gap is drastically decreased in these systems (28.7 and 4.0 kcal mol^−1^ for the silicon and phosphorus analogues, respectively). On the other hand, fusing the cluster with a cyclopentadiene unit (12) causes the 3c4e bond to become no longer available. Astonishingly, the system still obeys the regulatory effect by destabilizing the closed shell singlet system; therefore, the triplet state becomes more stable (by 11.4 kcal mol^−1^).

## Conclusions

In conclusion, it was demonstrated that the magnetic field induced by the carborane has a noticeable impact in a huge radius around the cluster. Moreover, this phenomenon solely offers an explanation for the difference between the NICS values of the five- and six-membered *exo* rings, thus showing the inadequacy of this descriptor to estimate the aromaticity of these systems. Our study concluded that the 2D aromatic properties of the investigated carborane fused five-membered rings are very similar to benzocarborane and can definitely be considered as a 2D non-aromatic compound; therefore, their aromatic character was significantly overestimated in earlier studies. The bonding situation of the *exo* ring strongly depends on the position of the heteroatom, and the symmetric carborane fused systems showed shorter bonds and higher Wiberg indices around the heteroatom compared to their oligo-aromatic counterparts, promoting the 3c4e bonding structure in the C–X–C moieties while forcing C_c_–C to become a single bond. This effect turned out to be strong enough to planarize phosphorus and silicon in C–P–C and C–Si–C analogue systems. The appearance of this phenomenon is also a sign of hindered conjugation; nevertheless, various isodesmic reactions were applied which showed no aromatic stabilization in the investigated systems. As decisive proof, fusing the system with a cyclopentadiene unit or squeezing the benzene ring between two carborane systems encouraged the formation of biradicaloid systems. These results will shed further light on the electronic structure of these compounds and should inspire the community to refine the theoretical approach and the practical applications of carborane-fused systems.

## Data availability

The data sets supporting this article have been uploaded as part of the ESI.‡

## Author contributions

Z. K supervised this study. Z. K. and D. B. conceptualized the work. D. B. wrote the original draft and edited the visuals. M. K. created the original visuals and arranged the ESI. M. K., E. H. and Z. H.-P. performed DFT calculations. Z. K., M. K., E. H. and Z. H.-P. edited the draft.

## Conflicts of interest

There are no conflicts to declare.

## Supplementary Material

SC-013-D2SC03511A-s001

SC-013-D2SC03511A-s002
