# Supplementary material for: Conjugation between 3D and 2D aromaticity: does it really exist? The case of carborane-fused heterocycles
Source: Chem Sci. 2022 Sep 6;13(38):11388–93. doi: 10.1039/d2sc03511a (PMC9533420; doi:10.1039/d2sc03511a)
Supplement: SC-013-D2SC03511A-s001 [file SC-013-D2SC03511A-s001.pdf]

Electronic Supplementary Material (ESI) for Chemical Science.
This journal is © The Royal Society of Chemistry 2022

Supporting Information

Conjugation between 3D and 2D aromaticity: does it really exist? The case of carborane-fused heterocycles

Dániel Buzsáki, Máté Barnabás Kovács, Evelyn Hümpfner, Zsófia Harcsa-Pintér, Zsolt Kelemen

Table of Contents

Computational details 2
Additional Tables and Figures 3
XYZ Geometries and Energies 13
  benzene, heterocylces, ortho-carborane and ethylene 13
  Carborane-Fused systems 14
  Benzene condensed compounds 19
  Triplet state compounds 21
  ISE compounds (Figure 3) 26

Computational details

All calculations have been carried out with Gaussian 09 program package.[1] Full geometry optimization was performed for all molecules at B3LYP/6-311+G** level of theory and single point calculations were carried out at B3LYP/cc-pVTZ level of theory. Despite this method was successfully used for carborane systems in previous studies,[2] linear-scaling local natural orbital coupled-cluster calculations (LNO-CCSD(T)) embedded in the MRCC program package[3] were also applied on some of the investigated systems (Table S6), which verified the accuracy of the applied DFT method. Harmonic vibrational frequencies were calculated at that level which was used for the optimization, to establish the nature of the stationary points obtained, as characterized by zero negative eigenvalues of the Hessian for minima. Open-shell systems were reoptimized at the same level, therefore adiabatic singlet-triplet gaps were calculated. For visualization, MOLDEN[4] and IQmol[5] programs were used. For the determination of bond critical points, Multiwfn program was used.[6] NBO 7.0[7] program was applied to calculate Wiberg indices and resonance structures based on the Natural Resonance Theory (NRT). The current density susceptibility induced by C-N-C *3c4e* bond delocalization in system **1'** was calculated by using the GIMIC 2.1.4 (gauge-including magnetically induced currents) program.[8] These results were visualized with the ParaView 5.0.10 program[9] using the line integral convolution (LIC) technique including arrows as the flow of the current.

The 2D aromaticity of the condensated system fused to the carborane was estimated by different parameters. The aromatic stabilization energy (ASE) is an energy-based descriptor for aromaticity. This value can be defined by calculating the isodesmic reaction energy of specific reactions, where any other stabilizing/destabilizing effects (ring strain, conjugation of the aliphatic systems) must be negligible. The nucleus independent chemical shift (NICS) is a magnetic-based measurement,[10] which case the negative of the isotropic tensor of the magnetic shielding is calculated in some particular points of the system. π-aromaticity is often defined by the NICS(1) descriptor, where the chosen point is 1 Å above the center of the ring plane. The 3D-aromaticity of the carborane was characterized by selecting the geometrical center of the cluster moiety ($NICS_c$). These values can be calculated by performing NMR calculations in the Gaussian program package.

Additional Tables and Figures

Table S1. Tabulated NICS(1), NICS(0) and (in case of the fused systems) NICS values in the geometrical center of the cluster ($NICS_c$) in ppm on B3LYP/cc-pVTZ//B3LYP/6-311+G** level of theory. The results for the investigated systems are compared to their pure aromatic heterocyclic counterparts. As $NICS(1)_{zz}$ values support the similar trend, NICS(1) can be safely applied as a magnetic descriptor for these compounds.

	carborane fused systems				heterocyclic systems		
	NICS(1) [ppm]	$NICS(1)_{zz}$ [ppm]	NICS(0) [ppm]	$NICS_c$ [ppm]	NICS(1) [ppm]	$NICS(1)_{zz}$ [ppm]	NICS(0) [ppm]
1	-5.0	-9.8	-8.6	-27.8	-10.5	-31.8	-14.0
1’	-7.0	-18.6	-11.4	-27.3			
2	-4.2	-7.2	-8.8	-27.2	-10.8	-29.4	-13.3
2’	-6.9	-15.5	-9.6	-27.3			
3	-4.9	-8.5	-8.6	-28.2	-9.6	-28.0	-12.0
3’	-8.3	-21.2	-11.3	-27.0			
4	-1.5	5.4	0.4	-23.9	11.8	-38.5	20.4
4’	2.6	-15.7	6.5	-19.4			
5	-2.6	-1.5	0.1	-27.7	-10.2	-29.2	-6.9
5’	-3.1	-2.7	-0.7	-27.7			
I	-2.5	-2.0	-1.3	-27.4	-10.4	-29.7	-8.2

Table S2 Reported ^{1}H NMR chemical shifts of the investigated systems

	**Compound**	**^{1}H NMR chemical shift (ppm)**
Previously known **non-aromatic** molecules	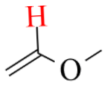	6.49[11]
	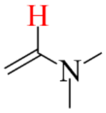	5.90[12]
	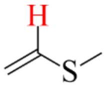	6.46[13]
Previously known **aromatic** molecules	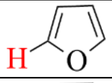	7.42[14]
	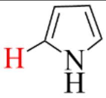	6.71[14]
	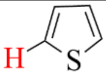	7.31[14]
Molecules reported by Xie et al.	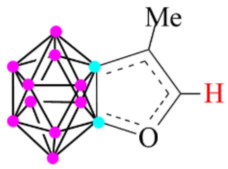	6.58[15]
	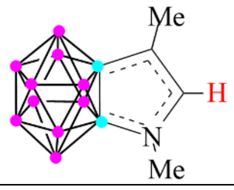	6.05[15]
	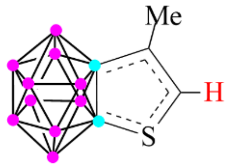	6.27[15]

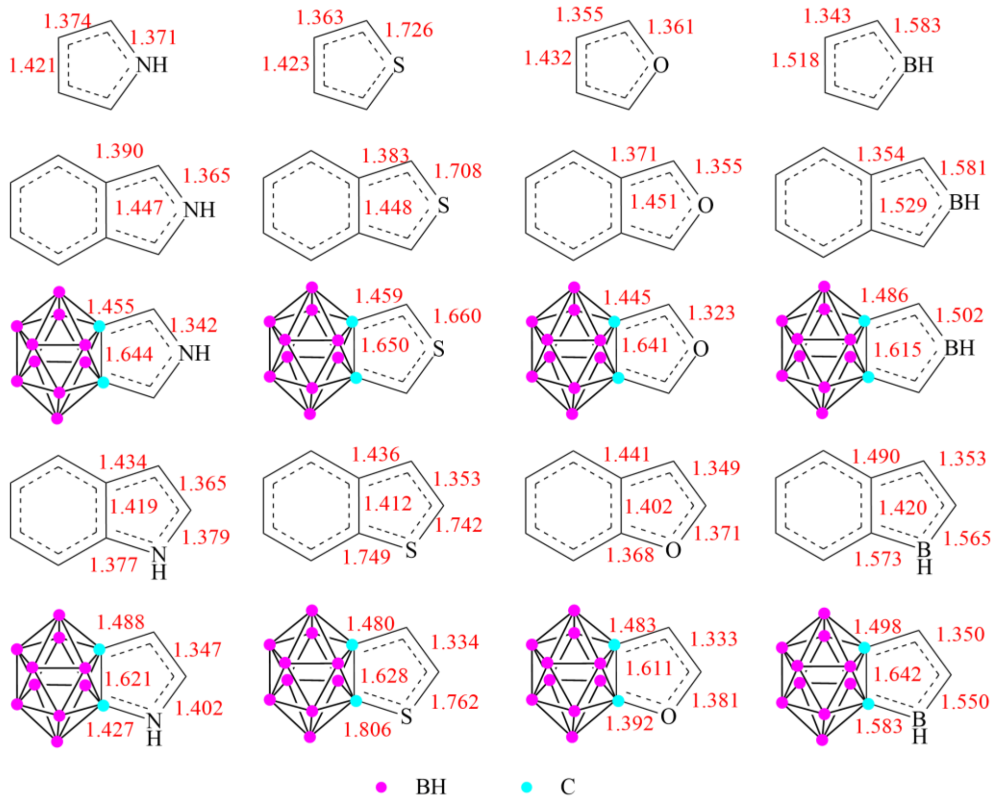

Fig. S1. Bond lengths (in Å) in the investigated systems (B3LYP/6-311+G** level of theory)

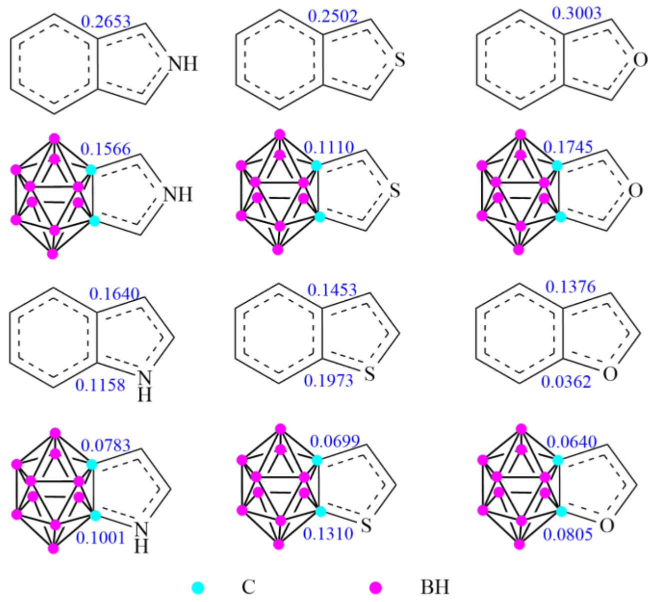

Fig. S2 Ellipticity of electron density (in a.u.) in the critical points of the bonds between the cluster atom and the *exo* ring in case of systems **1-3** and **1'-3'** compared to their respective oligoaromatic counterparts. Values are calculated at the B3LYP/6-311+G** level of theory.

Table S3 Dominant resonance structures (with weights higher than 5%) based on Natural Resonance Theory (NRT) calculations at the B3LYP/6-311+G** level of theory.[16]

**6**		**6’**	
Resonance structure	Weight (%)	Resonance structure	Weight (%)
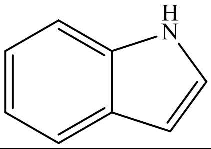	12.16	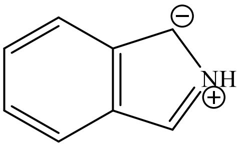	18.9
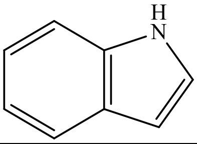	11.26	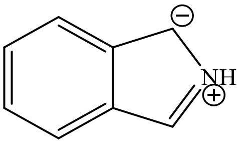	12.86
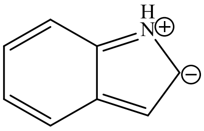	10.45	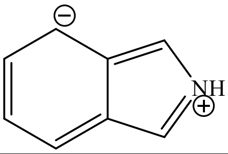	12.40
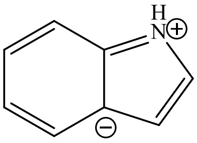	7.50	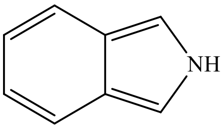	10.39
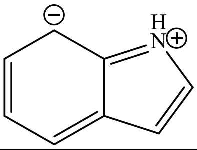	5.29	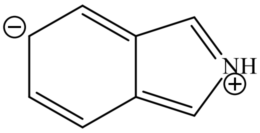	7.20

I **1** **1’**

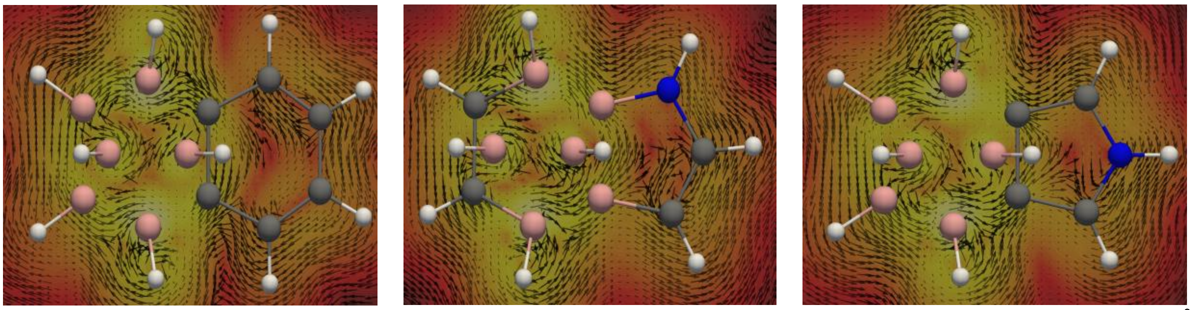

Fig. S3 The calculated current density (brighter colors show higher values) for **I**, **1** and **1’** 1 Å above the symmetry plane of the molecules visualized by a line integral convolution plot with arrows of the current flow, using the GIMIC program. In case of **1’**, the current density is definitely higher (depicted as brighter yellow) around the C-N-C system of the *exo* ring than in case of the other systems, showing the *3c4e* delocalization in the symmetric five-membered ring.

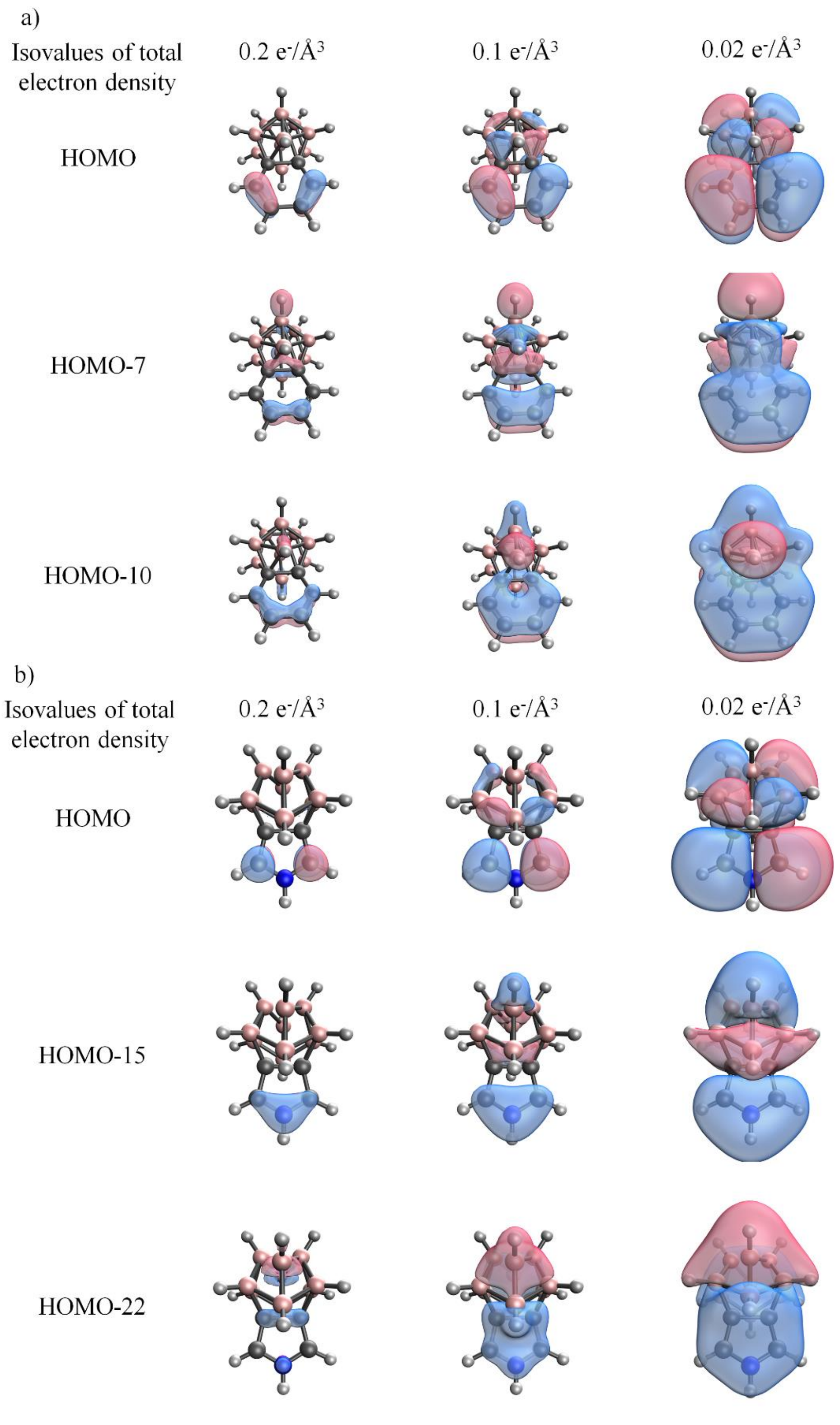

Fig. S4 Most important π-type Kohn-Sham orbitals of a) **I** and b) **1'**, visualized with different isovalues of total electron density at the B3LYP/cc-pVTZ//B3LYP/6-311+G** level of theory. The shape of orbitals at higher isovalues (0.2 $e^-/Å^3$ and 0.1 $e^-/Å^3$) shows that π-orbitals are mainly delocalized within the *exo* system, while lower isovalues (0.02 $e^-/Å^3$) can drastically change the message derived from the visualization. Moreover, all π-type orbitals of **I** and **1'** can be matched to each other, thus the π-system of the *exo* rings are similar in both cases.

Table S4 Tabulated data about the NICS values of the carborane along and 1 Å away from the perpendicular bisector line of the C-C bond at the B3LYP/cc-pVTZ// B3LYP/6-311+G** level of theory. The **d** values show the distance from the middle point of the C-C bond outside of the cluster (in Å).

d (Å)	NICS(0) [ppm]	NICS(1) [ppm]	d (Å)	NICS(0) [ppm]	NICS(1) [ppm]
0.0	-61.7	-29.9	1.9	-1.0	-0.6
0.1	-58.9	-28.2	2.0	-0.9	-0.6
0.2	-53.6	-25.4	2.1	-0.7	-0.5
0.3	-46.6	-22.1	2.2	-0.6	-0.4
0.4	-39.0	-18.6	2.3	-0.5	-0.4
0.5	-31.4	-15.3	2.4	-0.5	-0.3
0.6	-25.1	-12.3	2.5	-0.4	-0.3
0.7	-19.6	-9.7	2.6	-0.4	-0.3
0.8	-15.1	-7.6	2.7	-0.3	-0.3
0.9	-11.6	-5.9	2.8	-0.3	-0.2
1.0	-8.8	-4.5	2.9	-0.3	-0.2
1.1	-6.7	-3.5	3.0	-0.2	-0.2
1.2	-5.1	-2.7	3.1	-0.2	-0.2
1.3	-4.0	-2.1	3.2	-0.2	-0.2
1.4	-3.1	-1.7	3.3	-0.2	-0.2
1.5	-2.4	-1.3	3.4	-0.2	-0.1
1.6	-1.9	-1.1	3.5	-0.2	-0.1
1.7	-1.5	-0.9	3.6	-0.2	-0.1
1.8	-1.3	-0.8	3.7	-0.1	-0.1

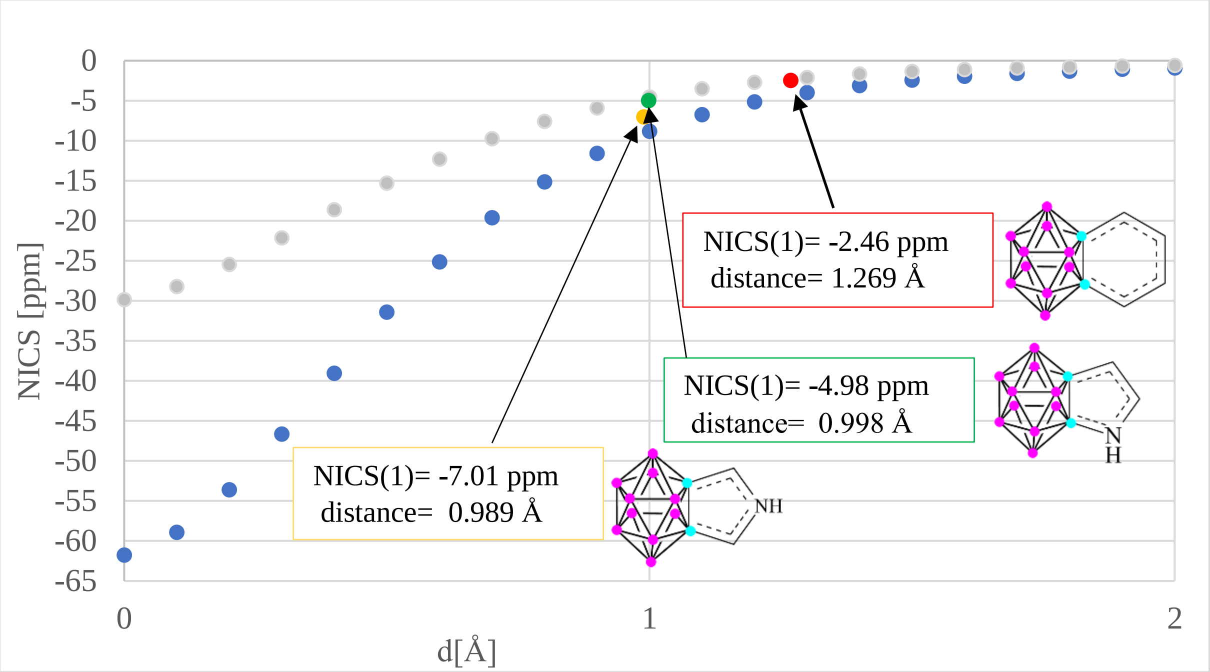

Fig. S5 The magnetic shielding of ghost atoms along (depicted as blue points) and 1 Å away (depicted as gray points) from the perpendicular bisector line of the C_C-C_C bond of the carborane (at B3LYP/cc-pVTZ//B3LYP/6-311+G** level of theory). The **d** values show the distance from the middle point of the C-C bond outside of the cluster (in Å).The NICS(1) results of **1** and **I** can perfectly fit on the calculated curve, while **1'** shows slight difference due to C-N-C *3c4e* bond delocalization.

Table S5 Singlet-triplet gap of all investigated compounds at the B3LYP/6-311+G** level of theory

	$\Delta E_{s\text{-}t}$ (kcal/mol)
**1**	72.1
**2**	66.0
**3**	72.9
**4**	61.0
**5**	71.8
**1'**	35.8
**2'**	22.2
**3'**	21.6
**4'**	10.7
**5'**	53.8
**I**	53.5
**7**	25.7
**8**	11.0
**9**	-11.6
**10**	28.7
**11**	4.0
**12**	-11.4

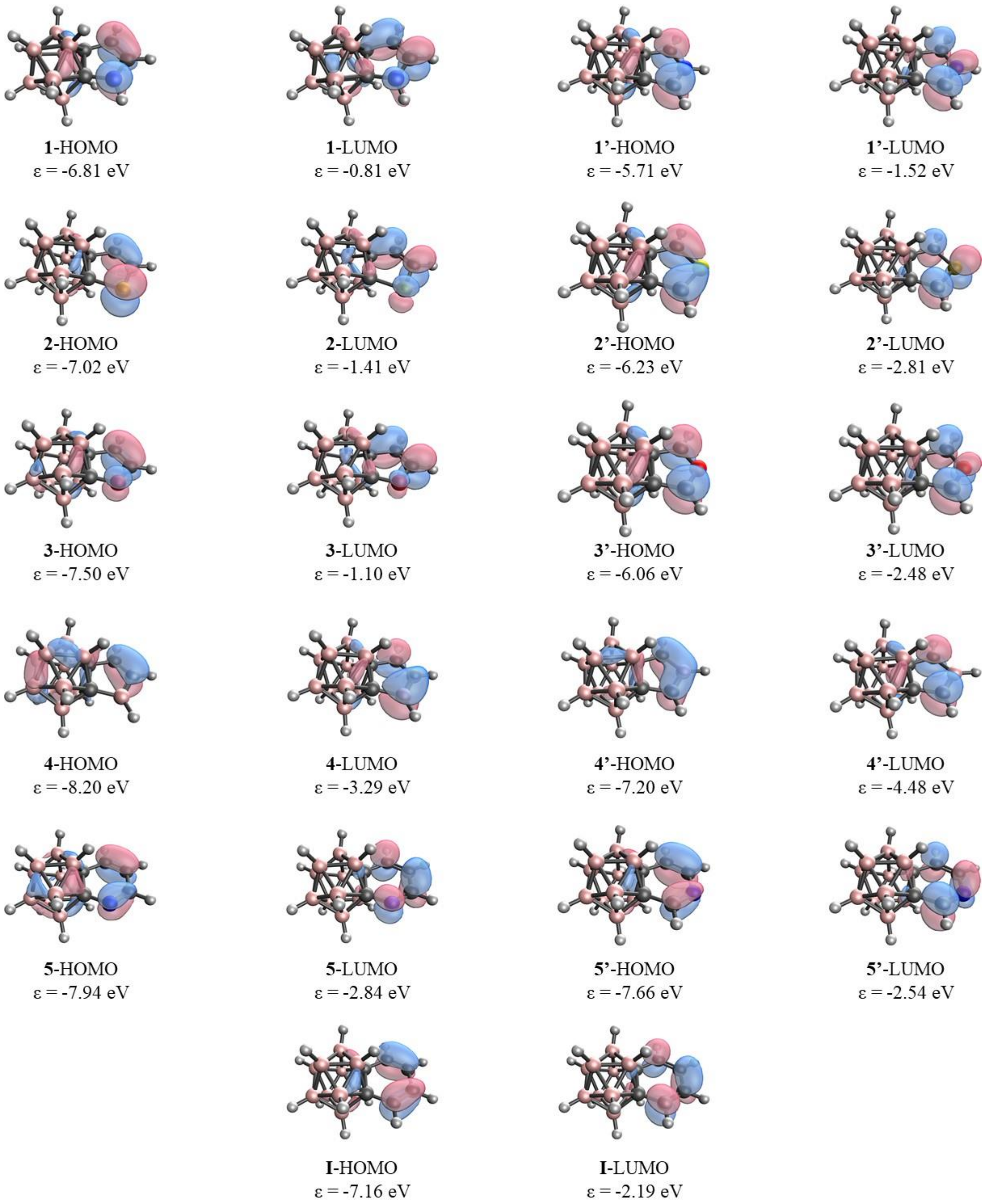

Fig. S6 The Kohn-Sham HOMO and LUMO of the investigated systems **1-5**, **1’-5’** and **I** (at the B3LYP/cc-pVTZ//B3LYP/6-311+G** level of theory)

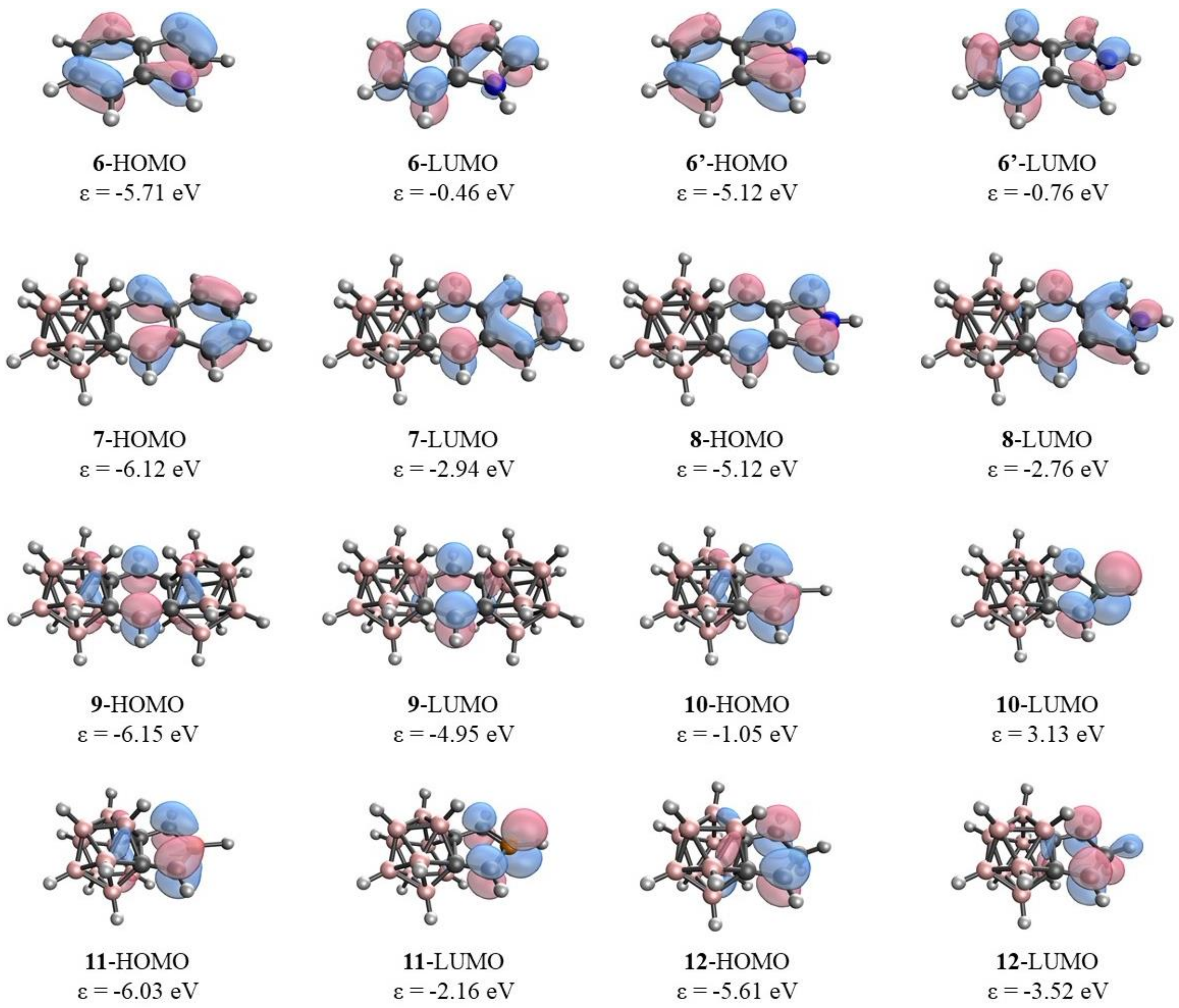

Fig. S7 The Kohn-Sham HOMO and LUMO of the investigated systems **6-12** and **6'** (at the B3LYP/cc-pVTZ//B3LYP/6-311+G** level of theory)

Table S6 Comparison of the energy differences at DFT and CC level of theories

	LNO-CCSD(T)/6-311+G** (kcal/mol)	B3LYP/6-311+G** (kcal/mol)	ΔΔE (kcal/mol)
$\Delta E_{(\mathbf{1\text{-}1'})}$	-22.4	-21.6	0.8
$\Delta E_{(\mathbf{2\text{-}2'})}$	-31.4	-31.8	0.4
$\Delta E_{(\mathbf{3\text{-}3'})}$	-36.8	-36.8	0.0
$\Delta E_{(\mathbf{4\text{-}4'})}$	-38.4	-39.9	1.5
$\Delta E_{(\mathbf{5\text{-}5'})}$	0.4	0.7	0.3

References

1. Frisch, M. J.; Trucks, G. W.; Schlegel, H. B.; Scuseria, G. E.; Robb, M. A.; Cheeseman, J. R.; Scalmani, G.; Barone, V.; Mennucci, B.; Petersson, G. A.; Nakatsuji, H.; Caricato, M.; Li, X.; Hratchian, H. P.; Izmaylov, A. F.; Bloino, J.; Zheng, G.; Sonnenberg, J. L.; Hada, M.; Ehara, M.; Toyota, K.; Fukuda, R.; Hasegawa, J.; Ishida, M.; Nakajima, T.; Honda, Y.; Kitao, O.; Nakai, H.; Vreven, T.; Montgomery, J. A. J.; Peralta, J. E.; Ogliaro, F.; Bearpark, M.; Heyd, J. J.; Brothers, E.; Kudin, K. N.; Staroverov, V. N.; Keith, T.; Kobayashi, R.; Normand, J.; Raghavachari, K.; Rendell, A.; Burant, J. C.; Iyengar, S. S.; Tomasi, J.; Cossi, M.; Rega, N.; Millam, J. M.; Klene, M.; Knox, J. E.; Cross, J. B.; Bakken, V.; Adamo, C.; Jaramillo, J.; Gomperts, R.; Stratmann, R. E.; Yazyev, O.; Austin, A. J.; Cammi, R.; Pomelli, C.; Ochterski, J. W.; Martin, R. L.; Morokuma, K.; Zakrzewski, V. G.; Voth, G. A.; Salvador, P.; Dannenberg, J. J.; Dapprich, S.; Daniels, A. D.; Farkas, O.; Foresman, J. B.; Ortiz, J. V.; Cioslowski, J.; Fox, D. J. Gaussian 09, Revision E.01. *Gaussian, Inc.: Wallingford, CT.*, 2013.
2. (a) Poater, J.; Solà, M.; Viñas, C.; Teixidor, F., *Chem. Eur. J.*, 2013, **19**, 4169–4175. (b) Poater, J.; Solà, M.; Viñas, C.; Teixidor, F., *Angew. Chem. Int. Ed.,* 2014, **53**, 12191–12195. (c) Poater, J.; Solà, M.; Viñas, C.; Teixidor, F., *Chem. Eur. J.,* 2016, **22**, 7437–7443. (d) Poater, J.; Viñas, C.; Olid, D.; Solà, M.; Teixidor, F., *Angew. Chem. Int. Ed.*, 2022. (e) Poater, J.; Viñas, C.; Bennour, I.; Escayola, S.; Solà, M.; Teixidor, F., *J. Am. Chem. Soc.*, 2020, **142**, 9396–9407.
3. (a) Kállay, M.; Nagy, P. R.; Mester, D.; Rolik, Z.; Samu, G.; Csontos, J.; Csóka, J.; Szabó, P. B.; Gyevi-Nagy, L.; Hégely, B.; Ladjánszki, I.; Szegedy, L.; Ladóczki, B.; Petrov, K.; Farkas, M.; Mezei, P. D.; Ganyecz, Á. MRCC, a Quantum Chemical Program Suite. Available at: Www.Mrcc.Hu. (b) Kállay, M.; Nagy, P. R.; Mester, D.; Rolik, Z.; Samu, G.; Csontos, J.; Csóka, J.; Szabó, P. B.; Gyevi-Nagy, L.; Hégely, B.; Ladjánszki, I.; Szegedy, L.; Ladóczki, B.; Petrov, K.; Farkas, M.; Mezei, P. D.; Ganyecz, Á. The MRCC Program System: Accurate Quantum Chemistry from Water to Proteins. *J. Chem. Phys.*, 2020, **152**.
4. Schaftenaar, G.; Noordik, J. H., *J. Comput. Mol. Des.*, 2000, **14**, 123–134.
5. Gilbert, A. T. B. IQmol Molecular Viewer. Available at: Http://Iqmol.Org.
6. Lu, T.; Chen, F., *J. Comput. Chem.*, 2012, **33**, 580–592.
7. NBO 7.0. E. D. Glendening, J. K. Badenhoop, A. E. Reed, J. E. Carpenter, J. A. Bohmann, C. M. Morales, P. Karafiloglou, C. R. Landis, and F. Weinhold, Theoretical Chemistry Institute, University of Wisconsin, Madison, WI (2018)
8. (a) J. Juselius, D. Sundholm, J. Gauss, *J. Chem. Phys.* 2004, **121**, 3952-3963. (b) H. Fliegl, S. Taubert, O. Lehtonen, D. Sundholm, *Phys. Chem. Chem. Phys.* 2011, **13**, 20500-20518.
9. Ahrens, James, Geveci, Berk, Law, Charles, ParaView: An End-User Tool for Large Data Visualization, Visualization Handbook, Elsevier, **2005**, ISBN-13: 978-0123875822
10. Afonin, A. V., *Rus. J. Org. Chem.*, 2011, **47**, 496–499.
11. Hall, H. K. Jr; Abdelkader, M; Glogowski, M. E., *J. Org. Chem.*, 1982, **47**, 3691–3694.
12. Aitken, A. R.; Armstrong, J. M.; Drysdale, M. J.; Ross, F. C.; Ryan, B. M., *J. Chem. Soc., Perkin Trans. 1,* 1999**,** **5**, 593–604.
13. Raymond , A. J.; Reid, M., *J. Chem. Soc., Perkin Trans. 2,* 2002, **6**, 1081–1091.
14. Qiu, Z.; Ren, S.; Xie, Z., *Acc. Chem. Res*, 2011, **44**, 299–309.
15. Similar calculations at other level of theory were performed in: Pino-Rios,R; Solà, M., *J. Phys. Chem. A*, 2021, **125**, 230−234.

XYZ Geometries and Energies

benzene, heterocylces, ortho-carborane and ethylene

Benzene
E(B3LYP/6-311+G**) = -232.311246037
C -0.00000000 -1.39462300 0.00000000
C 1.20790500 -0.69721800 -0.00000000
C 1.20780700 0.69730800 -0.00000000
C -0.00014600 1.39462500 -0.00000000
C -1.20784000 0.69733000 0.00000000
C -1.20773300 -0.69743300 0.00000000
H 2.14679200 1.23977300 -0.00000000
H -0.00004800 2.47904100 -0.00000000
H -2.14704500 1.23940800 0.00000000
H -2.14689500 -1.23959400 0.00000000
H 0.00025900 -2.47904100 0.00000000
H 2.14698200 -1.23951700 -0.00000000
pyridine
E(B3LYP/6-311+G**) = -248.3512114
N 0.00000000 0.00000000 1.41222700
C -0.00000000 1.13993000 0.71953100
C -0.00000000 1.19359600 -0.66970800
C -0.00000000 -0.00000000 -1.37901700
C -0.00000000 -1.19359600 -0.66970800
C -0.00000000 -1.13993000 0.71953100
H -0.00000000 2.05349100 1.30341800
H -0.00000000 2.14801900 -1.17768900
H -0.00000000 -0.00000000 -2.46081000
H -0.00000000 -2.14801900 -1.17768900
H -0.00000000 -2.05349100 1.30341800
pyrrole
E(B3LYP/ 6-311+G**) = -210.2305243
N -0.00000000 1.11894900 -0.00000000
C 1.12170200 0.33102000 0.00000000
C 0.71082300 -0.97994300 0.00000000
C -0.71041700 -0.98035900 -0.00000000
C -1.12232100 0.32999900 -0.00000000
H -2.10733300 0.76175500 -0.00000000
H -1.35592400 -1.84203200 -0.00000000
H 1.35738600 -1.84070000 0.00000000
H 2.10723400 0.76182600 0.00000000
H -0.00008600 2.12220800 -0.00000000
furan
E(B3LYP/ 6-311+G**) = -230.0878511
O 1.10293400 -0.34869700 -0.00000000
C 0.65941500 0.93850600 0.00000000
C -0.69535600 0.97050500 0.00000000
C -1.12695500 -0.39443600 0.00000000
C 0.00000000 -1.14692600 -0.00000000
H 0.18514400 -2.20542700 -0.00000000
H -2.13854700 -0.76134300 0.00000000
H -1.31212900 1.85227400 0.00000000
H 1.41943200 1.69816900 -0.00000000
thiophene
E(B3LYP/6-311+G**) = -553.0731543
S 0.00000000 1.19249800 0.00000000
C -1.23802400 -0.00945400 -0.00000000
C -0.71145000 -1.26711900 -0.00000000
C 0.71145500 -1.26711600 -0.00000000
C 1.23801800 -0.00944400 0.00000000
H 2.27441900 0.28169200 0.00000000
H 1.31524100 -2.16227200 -0.00000000
H -1.31523300 -2.16227700 -0.00000000
H -2.27442400 0.28168000 -0.00000000
borole
E(B3LYP/6-311+G**) = -180.266766997
C -1.25259300 0.34964800 -0.00003200
C -0.75910300 -0.89937500 0.00001800
C 0.75910300 -0.89937500 0.00001800
C 1.25259300 0.34964800 -0.00003300
B 0.00000000 1.31695900 0.00004500
H 2.31137300 0.57521900 -0.00011600
H 1.33159700 -1.82244200 0.00003400
H -1.33159700 -1.82244200 0.00003100
H -2.31137300 0.57521900 -0.00011600
H -0.00000000 2.50637400 0.00011600
phosphole
E(B3LYP/6-311+G**) = -496.812660912
P -1.21996100 -0.00001800 -0.12063700
C 0.04959500 -1.28583100 0.04847900
C 1.28258200 -0.72891000 0.00218500
C 1.28273600 0.72890200 0.00209900
C 0.04970800 1.28579700 0.04817400
H -0.15535700 2.34775300 0.04124700
H 2.20309700 1.30199700 -0.03147200
H 2.20274900 -1.30227200 -0.03288600
H -0.15529800 -2.34781000 0.04131200
H -1.78349700 0.00085100 1.18572700
silacyclopentadienyl anion
E(B3LYP/6-311+G**) = -444.970251228
C 0.03859000 -1.32330800 0.07826000
Si -1.27772800 0.00023600 -0.14512000
C 0.03914400 1.32341900 0.07866600
C 1.27498900 0.72470600 0.00665100
C 1.27472900 -0.72517800 0.00662900
H -0.05837500 2.40679900 0.05253200
H 2.21396300 1.27497500 -0.08547400
H 2.21352700 -1.27581300 -0.08506500
H -0.05947300 -2.40666400 0.05335000
H -2.18615400 -0.00043500 1.07509800
Ethylene
E(B3LYP/6-311+G**) = -78.6155123660
C -0.00000000 0.00000000 0.66448100
C 0.00000000 0.00000000 -0.66448100
H 0.00000000 0.92265200 1.23482800
H 0.00000000 0.92265200 -1.23482800
H -0.00000000 -0.92265200 -1.23482800

H -0.00000000 -0.92265200 1.23482800
ortho-carborane
E(B3LYP/6-311+G**) = -332.163886444
C -0.00827500 -0.81409200 -1.27308900
C -0.00206300 0.81114400 -1.27496600
B 0.89793900 1.43688100 0.02102200
B 1.45734700 -0.00428600 0.90413900
B 0.00277100 -0.88985700 1.46306400
B -1.45005400 0.00639500 0.91592300
B 0.00922100 0.89333600 1.46108000
B -0.88684600 1.44337800 0.02777100
B -1.46053400 0.00426100 -0.84761800
B -0.89751900 -1.43682500 0.03145300
B 0.88727300 -1.44332600 0.02412400
B 1.45275700 -0.00618200 -0.85922800
H -0.00421200 1.28640300 -2.24456400
H 2.48337800 -0.00765500 1.48941600
H -2.47093900 0.01090400 1.51011000
H 0.00483300 -1.53048100 2.45599900
H 0.01586300 1.53606700 2.45261900
H -2.33773700 0.00668400 -1.63138600
H -1.46954800 2.45532700 -0.13692100
H -1.48742800 -2.44492800 -0.13120100
H 1.46882100 -2.45551600 -0.14305400
H 2.32402300 -0.01075500 -1.65091700
H 1.48684600 2.44436500 -0.14886000
H -0.01364900 -1.29159700 -2.24156800

Carborane-Fused systems

1
E(B3LYP/6-311+G**) = -463.727922908
E(LNO-CCSD(T)/6-311+G**) = -462.369678389
C -0.62803900 0.84461800 -0.00101700
C -0.64684300 -0.77538900 -0.02316800
N -2.02925100 -1.11826300 -0.07210700
C -2.79834700 0.04748300 0.00185400
C -2.07581500 1.18226500 0.00113200
B 0.63116300 -1.44568600 0.88257900
B 2.09242100 -0.92862500 0.00415000
B 2.13080900 0.87290400 0.02421400
B 1.60156500 0.00050100 -1.43526500
B 0.70828200 1.45358600 -0.87907300
B 0.70321100 1.44045400 0.91587600
B 1.57158800 -0.02972500 1.45576800
B -0.16103500 0.01541600 1.47785300
B 0.66056200 -1.43000200 -0.91821300
B -0.13558100 0.03402400 -1.48975700
H -2.33343000 -1.96485300 0.38610600
H 3.07041400 -1.59201200 0.01034700
H -0.92476900 0.05423700 -2.36042000
H -0.96685800 0.01357100 2.33470200
H 2.15414700 -0.05710800 2.48362100
H 0.44029500 -2.46383200 1.45014000
H 0.56941600 2.45386200 1.50518900
H 0.58402000 2.47582200 -1.45480900
H 2.20396900 0.00239100 -2.45183000
H 0.48176100 -2.43663600 -1.50702700
H 3.13880200 1.48946600 0.03823100
H -3.87480100 -0.04438500 0.01770600
H -2.45886800 2.18922300 -0.01066400
1’
E(B3LYP/6-311+G**) = -463.693556736
E(LNO-CCSD(T)/6-311+G**) = -462.333939584
C 0.63801800 0.82361800 0.00000400
C 0.63801400 -0.82361100 0.00000300
C 2.05904500 -1.15249400 0.00000100
N 2.75368900 -0.00000300 -0.00000000
C 2.05905000 1.15249200 -0.00000600
B -0.68897200 -1.44187900 -0.90196500
B -0.68897300 -1.44188000 0.90197100
B 0.13973100 0.00000200 1.48320600
B -0.68896800 1.44188700 0.90196200
B -0.68897100 1.44188100 -0.90197700
B 0.13972900 -0.00000700 -1.48320400
B -1.59769600 -0.00000600 -1.44152100
B -2.12147000 -0.90539600 0.00000700
B -1.59769000 0.00000700 1.44152300
B -2.12147200 0.90539300 -0.00000500
H -3.11590300 -1.54457000 0.00001100
H 0.93124600 0.00001100 2.35567800
H 0.93124200 -0.00001200 -2.35567800
H -2.18335100 -0.00001600 -2.46844100
H -0.53902300 -2.45739400 -1.48513000
H -0.53903100 2.45739200 -1.48514900
H -0.53902900 2.45739900 1.48513200
H -2.18334700 0.00000600 2.46844200
H -0.53902000 -2.45738700 1.48514800
H -3.11590600 1.54456600 0.00000000
H 2.52939200 2.11831300 0.00000600
H 2.52938300 -2.11831800 -0.00001000
H 3.76651700 -0.00000400 0.00000100
2
E(B3LYP/6-311+G**) = -806.576082337
E(LNO-CCSD(T)/6-311+G**) = -804.827232456
C -1.64539100 1.59197500 -0.00007200
C -0.29834100 0.96924000 -0.00004900
C -0.50346600 -0.64922200 0.00000800
S -2.28197000 -0.99593900 -0.00002100
C -2.66872900 0.73024600 0.00007400
B 0.06478200 0.09284400 -1.47533600
B 1.08899400 1.41185100 -0.89484500
B 1.08896800 1.41191400 0.89475200
B 1.79280500 -0.13604100 1.44691000
B 0.70929600 -1.44905100 0.89728500
B 0.06473500 0.09294400 1.47531000
B 0.70931100 -1.44911600 -0.89718300
B 1.79284600 -0.13615100 -1.44686800
B 2.20501700 -1.09689500 0.00006100
B 2.44251500 0.68267500 0.00000100
H 3.10194900 -1.86616000 0.00010000
H -0.71546800 0.19518700 -2.34829000

H -0.71553600 0.19535300 2.34823700
H 2.37461200 -0.21379300 2.47225100
H 0.41280100 -2.42936600 1.48168400
H 1.06383900 2.43523200 1.48139900
H 1.06387200 2.43512500 -1.48156900
H 2.37468400 -0.21397500 -2.47218700
H 0.41284800 -2.42947700 -1.48151800
H 3.51204000 1.18471400 -0.00000700
H -3.71709800 0.99660700 0.00014100
H -1.75780300 2.66726800 -0.00010300
2'
E(B3LYP/6-311+G**) = -806.576914448
E(LNO-CCSD(T)/6-311+G**) = -804.777299913
C -0.38235600 0.82686800 0.00000200
C -0.38235500 -0.82686600 -0.00000000
C -1.77541300 -1.26949600 0.00000100
S -2.85673500 -0.00000100 0.00000000
C -1.77541400 1.26949600 0.00000100
B 0.94855900 -1.44133000 0.89895600
B 0.94855800 -1.44133000 -0.89895600
B 0.10866600 0.00000000 -1.47816200
B 0.94855800 1.44133100 -0.89895600
B 0.94855700 1.44132900 0.89895600
B 0.10866600 -0.00000100 1.47815900
B 1.85194000 -0.00000000 1.44163500
B 2.38141100 -0.90275900 0.00000100
B 1.85193700 -0.00000200 -1.44163300
B 2.38140700 0.90276100 -0.00000200
H 3.37422900 -1.54388400 -0.00000300
H -0.67477100 -0.00000300 -2.35630100
H -0.67476700 -0.00000300 2.35630200
H 2.43123300 0.00000300 2.47181800
H 0.79324800 -2.45413200 1.48466200
H 0.79324800 2.45413400 1.48465700
H 0.79324900 2.45413300 -1.48466200
H 2.43122800 0.00000100 -2.47181700
H 0.79324700 -2.45413200 -1.48466200
H 3.37422700 1.54388400 -0.00000100
H -2.11233500 2.29260500 0.00000200
H -2.11233300 -2.29260500 -0.00000300
3
E(B3LYP/6-311+G**) = -483.587250839
E(LNO-CCSD(T)/6-311+G**) = -482.201609851
C -0.63173700 0.85097800 -0.00000700
C -0.64587800 -0.76316200 0.00000100
O -1.98332800 -1.15587500 0.00000300
C -2.75993000 -0.01228300 0.00000100
C -2.08924600 1.14428000 -0.00000400
B 0.63286200 -1.43951000 0.90079100
B 0.63286600 -1.43951600 -0.90079200
B -0.16163700 0.02680200 -1.48404800
B 0.69510600 1.45367800 -0.89795300
B 0.69510400 1.45368100 0.89795000
B -0.16164000 0.02680800 1.48405100
B 1.57193800 -0.01326400 1.44724200
B 2.07904200 -0.92421000 0.00000400
B 1.57193800 -0.01326800 -1.44723700
B 2.11734200 0.87478400 -0.00000000
H 3.05677700 -1.58736800 0.00000600
H -0.96502000 0.03728600 -2.34184500
H -0.96502700 0.03729800 2.34184300
H 2.16461200 -0.02467100 2.46907900
H 0.44307400 -2.44962100 1.47742100
H 0.56710300 2.47079100 1.48135800
H 0.56710700 2.47078300 -1.48136800
H 2.16461400 -0.02468300 -2.46907400
H 0.44308200 -2.44962700 -1.47742000
H 3.12565100 1.49035300 -0.00000100
H -3.82118600 -0.20534600 0.00000400
H -2.51802300 2.13299800 -0.00000800
3'
E(B3LYP/6-311+G**) = -483.528570694
E(LNO-CCSD(T)/6-311+G**) = -482.142925962
C 0.64453300 0.82171400 -0.00001000
C 0.64453500 -0.82171700 -0.00000300
C 2.06205500 -1.12104200 0.00000000
O 2.76864000 0.00000100 0.00000100
C 2.06205400 1.12104200 0.00000200
B -0.67987300 -1.44377300 -0.90376800
B -0.67987600 -1.44377100 0.90376900
B 0.15156200 -0.00000100 1.48904500
B -0.67987700 1.44377300 0.90377400
B -0.67987200 1.44377400 -0.90376400
B 0.15156500 0.00000400 -1.48904000
B -1.58721400 0.00000100 -1.44201400
B -2.10983500 -0.90655700 -0.00000300
B -1.58721100 -0.00000200 1.44201100
B -2.10983400 0.90655600 0.00000100
H -3.10403700 -1.54510800 -0.00000300
H 0.94614700 0.00000000 2.35886100
H 0.94614400 0.00000600 -2.35886300
H -2.16918200 0.00000100 -2.47048500
H -0.52778800 -2.45913200 -1.48545900
H -0.52778900 2.45913600 -1.48544900
H -0.52779500 2.45913200 1.48546300
H -2.16917800 -0.00000700 2.47048200
H -0.52778700 -2.45913400 1.48545100
H -3.10403900 1.54510300 0.00000100
H 2.59472000 2.05356600 -0.00000100
H 2.59472400 -2.05356500 0.00000900
4
E(B3LYP/6-311+G**) = -433.826551144
E(LNO-CCSD(T)/6-311+G**) = -432.516361978
C -2.14895900 -1.08464700 -0.00002700
C -2.96272400 -0.00785900 0.00004600
B -2.14405200 1.30787800 0.00008900
C -0.61715500 0.88912100 0.00015000
B -0.19459400 0.04971200 1.46083600
B 0.73884200 1.44749200 0.88492900
B 0.73880800 1.44779600 -0.88466500

B -0.19462400 0.05015700 -1.46082400
B 0.58251300 -1.44911300 -0.88912900
B 2.04786700 -0.96880700 -0.00015800
B 1.54780700 -0.04341500 -1.45275200
B 2.14748400 0.81076500 0.00008300
B 1.54787900 -0.04377100 1.45272800
B 0.58262200 -1.44941900 0.88860200
C -0.68903000 -0.75088000 0.00001400
H 3.00794900 -1.65772100 -0.00028600
H -0.97135800 0.07197100 -2.34384700
H -0.97104500 0.07121100 2.34411200
H 2.13837900 -0.07807800 2.47547900
H 0.37113700 -2.44750700 1.48101400
H 0.63555900 2.45945300 1.48163900
H 0.63531600 2.46011100 -1.48072900
H 2.13815600 -0.07749200 -2.47560500
H 0.37085200 -2.44709300 -1.48166000
H 3.17717200 1.39027200 0.00022600
H -2.53153500 2.42609500 0.00003900
H -2.45534600 -2.12512500 -0.00013300
H -4.04079800 -0.11687400 -0.00004200
4’
E(B3LYP/6-311+G**) = -433.763044014
E(LNO-CCSD(T)/6-311+G**) = -432.45518296
C 2.11369400 1.18922400 -0.00055700
B 3.03156700 -0.00004200 -0.00002700
C 2.11364500 -1.18920200 0.00045800
C 0.67794900 -0.80771200 0.00044300
B 0.19606800 0.00146600 1.48169100
B -0.64394300 -1.45125200 0.88581900
B -0.64435900 -1.45297500 -0.88323300
B 0.19616500 -0.00150700 -1.48148200
B -0.64403200 1.45124700 -0.88584500
B -2.08636500 0.88821300 -0.00089800
B -1.53774300 -0.00142000 -1.45567100
B -2.08639200 -0.88816500 0.00091600
B -1.53765400 0.00143300 1.45569200
B -0.64418500 1.45298200 0.88322600
C 0.67798800 0.80770500 -0.00053000
H -3.08054500 1.52577500 -0.00150500
H 0.97696100 -0.00246000 -2.35649500
H 0.97734400 0.00237500 2.35623300
H -2.13665700 0.00242300 2.47363600
H -0.47944400 2.46162000 1.47079800
H -0.47920800 -2.45871400 1.47540900
H -0.47959900 -2.46149800 -1.47100000
H -2.13665300 -0.00239000 -2.47367100
H -0.47922300 2.45857500 -1.47564700
H -3.08059300 -1.52569900 0.00192900
H 2.34251600 -2.25100500 0.00118500
H 2.34244300 2.25107300 -0.00066000
H 4.21736000 -0.00006200 -0.00003100
5
E(B3LYP/6-311+G**) = -501.837241475
E(LNO-CCSD(T)/6-311+G**) = -500.380348897
C 0.45651900 -0.82939800 -0.00002100
C 0.42642500 0.85224700 0.00007200
C 1.77795900 1.44910200 0.00010000
C 2.87346300 0.67402800 -0.00007000
C 2.78315200 -0.78332700 0.00027000
N 1.70631300 -1.48182600 0.00001200
B -0.89585300 1.44507500 0.89191200
B -0.89582000 1.44518800 -0.89174500
B -0.01216500 0.00560700 -1.45324400
B -0.87031700 -1.44366200 -0.89135100
B -0.87035700 -1.44377700 0.89114200
B -0.01221700 0.00542000 1.45326300
B -1.76702600 -0.00807400 1.44512000
B -2.32476800 0.88557700 0.00002200
B -1.76697300 -0.00788400 -1.44517300
B -2.30933500 -0.90240700 -0.00009800
H -3.32188100 1.51934800 0.00005000
H 0.77215700 0.01598600 -2.32976300
H 0.77206000 0.01568500 2.32982400
H -2.34322500 -0.01169300 2.47655700
H -0.74136300 2.45322900 1.48652100
H -0.69838800 -2.44813000 1.48241900
H -0.69831700 -2.44794000 -1.48274600
H -2.34313100 -0.01137100 -2.47663300
H -0.74131300 2.45342300 -1.48621200
H -3.29623800 -1.55204500 -0.00016000
H 1.83875400 2.53129100 0.00007600
H 3.86075000 1.11979300 -0.00041400
H 3.71498300 -1.34601300 -0.00095500
5’
E(B3LYP/6-311+G**) = -501.836193208
E(LNO-CCSD(T)/6-311+G**) = -500.379671123
B 2.32347700 -0.89688900 -0.00000000
B 1.78140400 -0.00139400 1.44693100
B 0.88583800 -1.43589900 0.89150100
B 0.88584400 -1.43588800 -0.89151700
B 1.78141300 -0.00137900 -1.44692600
B 0.89251500 1.44316300 -0.89259900
C -0.43355500 0.83069300 0.00000300
C -1.78932800 1.41886400 0.00001600
C -2.88515200 0.64384600 0.00008900
N -2.89499000 -0.75968100 -0.00001500
C -1.79492000 -1.40936300 -0.00002000
C -0.43325000 -0.80271500 -0.00001000
B 0.02785300 0.00699100 -1.46903400
B 2.32579100 0.89417900 0.00000800
B 0.89250900 1.44315300 0.89261400
B 0.02784500 0.00697500 1.46903000
H 3.31883400 1.53490300 0.00001600
H -0.75641100 0.00908000 2.34528800
H -0.75639600 0.00910700 -2.34529600
H 2.36071800 -0.00278100 -2.47633800
H 0.73239500 2.45477300 -1.47822900
H 0.71080800 -2.44636600 -1.47524500
H 0.71079700 -2.44638300 1.47521600

H 2.36070000 -0.00280500 2.47634800
H 0.73238500 2.45475900 1.47825200
H 3.31285600 -1.54311900 0.00000100
H -1.86385300 2.49897800 -0.00018600
H -3.87138300 1.09166300 -0.00023200
H -1.83174200 -2.49704200 -0.00000100
I
E(B3LYP/6-311+G**) = -485.799322542
C 1.76954700 1.45493200 0.00000000
C 0.43048000 0.82337900 -0.00000300
C 0.43047900 -0.82338000 -0.00000100
C 1.76954800 -1.45493200 -0.00000100
C 2.89917300 -0.72720300 0.00000100
C 2.89917300 0.72720300 0.00000200
B -0.03720000 -0.00000500 1.46414300
B -1.79188900 -0.00000600 1.44538300
B -2.33543200 0.89530900 0.00000300
B -2.33542800 -0.89532000 0.00000200
B -1.79188800 -0.00000200 -1.44537500
B -0.03720700 0.00001200 -1.46414700
B -0.89911200 1.43666800 -0.89153400
B -0.89910700 1.43666300 0.89154200
B -0.89910000 -1.43666100 -0.89154100
B -0.89910300 -1.43666200 0.89153300
H -3.32561100 -1.54050500 -0.00000600
H 0.74124800 0.00000800 2.34555900
H 0.74124400 0.00000400 -2.34556100
H -2.37106900 0.00000200 -2.47538300
H -0.73507000 -2.44801600 -1.47765700
H -0.73508900 2.44802300 -1.47765300
H -0.73507900 2.44802100 1.47765300
H -2.37107600 0.00000000 2.47538700
H -0.73507100 -2.44802400 1.47763900
H -3.32561200 1.54049900 -0.00000100
H 1.78872800 -2.53784100 -0.00000400
H 3.85533100 -1.23774800 0.00000000
H 3.85533100 1.23774900 0.00000400
H 1.78872800 2.53784100 -0.00000200
7
E(B3LYP/6-311+G**) = -639.458335046
C 4.34781000 -0.72268300 0.00000000
C 4.34781000 0.72268300 0.00000000
C 3.18664100 1.41628800 0.00000000
C 1.90383100 0.73910300 -0.00000000
C 1.90383100 -0.73910300 -0.00000000
C 3.18664100 -1.41628800 0.00000000
C 0.74364600 1.44843900 -0.00000000
C -0.58418600 0.81938800 -0.00000000
B -1.05983700 -0.00000000 1.47102900
B -1.91760800 -1.43584900 0.89312900
B -3.35312900 -0.89752300 0.00000000
B -1.91760800 -1.43584800 -0.89312900
C -0.58418600 -0.81938800 -0.00000000
C 0.74364600 -1.44843900 -0.00000000
B -1.91760800 1.43584800 0.89312900
B -1.91760800 1.43584900 -0.89312900
B -1.05983700 0.00000000 -1.47102900
B -2.81338500 0.00000000 -1.44427300
B -3.35312900 0.89752300 0.00000000
B -2.81338500 -0.00000000 1.44427400
H -1.75333400 2.44850600 -1.47701800
H -4.34345200 -1.54320100 0.00000000
H -4.34345200 1.54320100 0.00000000
H -3.39152900 0.00000000 -2.47498000
H -1.75333400 -2.44850600 1.47701900
H -1.75333400 -2.44850600 -1.47701900
H -3.39152800 -0.00000000 2.47498000
H -1.75333400 2.44850600 1.47701900
H -0.28052200 0.00000000 -2.35205900
H -0.28052200 -0.00000000 2.35205800
H 0.76013700 2.53187300 -0.00000000
H 0.76013700 -2.53187400 -0.00000000
H 3.18740500 -2.50065900 0.00000000
H 5.29601100 -1.24772900 0.00000000
H 3.18740500 2.50065900 0.00000000
H 5.29601100 1.24772900 0.00000000
8
E(B3LYP/6-311+G**) = -617.360809776
N 4.21856500 0.00000000 -0.00000300
C 3.48829500 1.14082900 -0.00004900
C 2.12155900 0.74474000 0.00002000
C 2.12155900 -0.74474000 -0.00002500
C 3.48829400 -1.14082900 0.00006200
C 0.97290300 1.48055400 0.00004100
C -0.34038100 0.82717800 0.00002500
C -0.34038200 -0.82717800 -0.00003200
C 0.97290300 -1.48055400 -0.00005800
B -0.82502600 -0.00005000 1.47095300
B -1.68076200 1.43354300 0.89477500
B -2.57903900 -0.00004800 1.44184800
B -3.11649000 0.89942600 0.00003500
B -2.57904900 0.00004800 -1.44184000
B -1.68076900 1.43360200 -0.89467800
B -0.82503600 0.00004900 -1.47095800
B -1.68076900 -1.43354300 -0.89477300
B -3.11648900 -0.89942600 -0.00002400
B -1.68076200 -1.43360200 0.89468000
H 0.98161800 2.56312100 0.00004200
H 0.98161800 -2.56312100 -0.00005000
H 3.94636000 2.11395700 -0.00008000
H 5.22947100 0.00000000 -0.00000500
H -1.51976900 2.44678800 -1.47961600
H -4.10663200 -1.54594800 -0.00004200
H -4.10663200 1.54594800 0.00006100
H -3.15598300 0.00008200 -2.47378400
H -1.51976000 -2.44678800 1.47961500
H -1.51977000 -2.44669000 -1.47977800
H -3.15596600 -0.00008300 2.47379600
H -1.51975900 2.44669000 1.47977900
H -0.04933100 0.00008000 -2.35580900

H -0.04931400 -0.00008000 2.35579800
H 3.94636100 -2.11395600 0.00010600
9
E(B3LYP/6-311+G**) = -739.207142070
C 1.27531900 -1.04236300 -0.01606400
B 2.66752200 -1.46936300 -0.84085400
B 3.56194700 -0.00034200 -1.32795900
B 4.04353300 0.89057300 0.13845200
B 2.57436700 1.46916100 0.95144900
B 2.66748700 1.46878700 -0.84160400
C 1.27542800 1.04234200 -0.01636100
B 1.58267200 0.00045700 1.32121000
B 3.37016300 0.00033800 1.52989800
B 4.04348800 -0.89064100 0.13873700
B 2.57423200 -1.46882900 0.95219000
B 1.81762000 -0.00061600 -1.42427400
C -0.00013900 -1.55424500 -0.38179100
C -1.27549500 -1.04246500 -0.01616400
B -2.57439600 -1.46891100 0.95199400
B -1.58247600 0.00012700 1.32105500
B -2.57406200 1.46908200 0.95164100
B -2.66735400 1.46897000 -0.84139700
B -3.56209300 -0.00004900 -1.32783500
B -4.04337700 0.89077700 0.13864900
B -3.36998900 0.00023100 1.52995900
B -4.04360800 -0.89043700 0.13876600
B -2.66782400 -1.46919300 -0.84102300
B -1.81780600 -0.00040200 -1.42432000
C 0.00013300 1.55443100 -0.38193800
C -1.27525100 1.04239200 -0.01629000
H 5.03801700 1.52492800 0.20182900
H 2.60599100 -2.45108000 -1.49498300
H 0.90370800 0.00038000 2.26442400
H 2.41205100 -2.43371500 1.60904900
H 2.41256500 2.43462200 1.60756700
H 3.84884000 0.00073700 2.60928400
H 1.14874600 -0.00056900 -2.39045800
H 4.18202500 -0.00037800 -2.33358300
H 5.03788500 -1.52509500 0.20251900
H 2.60627600 2.45019100 -1.49621700
H -4.18223800 0.00001800 -2.33342000
H -3.84857200 0.00052600 2.60938400
H -5.03777800 1.52524500 0.20219700
H -5.03809400 -1.52475200 0.20254900
H -0.90344500 -0.00020600 2.26422900
H -2.41245900 -2.43393800 1.60870600
H -2.60619600 2.45047400 -1.49587000
H -1.14896300 -0.00029000 -2.39052200
H -2.60652400 -2.45087400 -1.49523200
H -2.41203900 2.43439500 1.60792400
H 0.00007500 2.16465900 -1.28075700
H -0.00006400 -2.16444300 -1.28063300
10
E(B3LYP/6-311+G**) = -698.503441511
C 1.80252800 1.38183700 -0.00000400
Si 2.88533800 0.00000000 0.00000100
C 1.80252800 -1.38183700 0.00000000
C 0.43486600 -0.84286100 -0.00000000
B -0.10552700 -0.00000100 1.46666800
B -0.93292800 -1.42999200 0.89742700
B -0.93292500 -1.42999100 -0.89742400
B -0.10552800 0.00000100 -1.46666800
B -0.93292900 1.42999200 -0.89742700
B -2.37550400 0.90520600 0.00000000
B -1.84958100 -0.00000100 -1.43483900
B -2.37550400 -0.90520600 0.00000100
B -1.84957900 0.00000100 1.43484000
B -0.93292500 1.42999100 0.89742400
C 0.43486600 0.84286100 -0.00000200
H -3.37215200 1.55152500 0.00000100
H 0.64579900 -0.00000100 -2.37315400
H 0.64580200 0.00000100 2.37315200
H -2.44283900 0.00000200 2.46382300
H -0.80371000 2.44840200 1.48830500
H -0.80371200 -2.44840300 1.48830800
H -0.80371200 -2.44840200 -1.48830600
H -2.44284300 -0.00000300 -2.46382100
H -0.80371400 2.44840200 -1.48830900
H -3.37215200 -1.55152500 0.00000000
H 1.95306100 -2.45031000 -0.00001300
H 1.95306000 2.45031000 0.00002500
H 4.36830800 0.00000000 -0.00000600
11
E(B3LYP/6-311+G**) = -750.260425060
C 1.77942700 1.34717600 0.00001900
P 2.76918200 -0.00000800 -0.00004700
C 1.77942100 -1.34717800 0.00004200
C 0.38923600 -0.83914400 0.00002000
B -0.10921600 -0.00002000 1.47238700
B -0.94699000 -1.43966200 0.89872300
B -0.94701900 -1.43967400 -0.89871300
B -0.10922500 -0.00001900 -1.47237900
B -0.94698400 1.43967000 -0.89873400
B -2.38244700 0.90178600 -0.00001100
B -1.85148900 0.00000900 -1.44055700
B -2.38248300 -0.90173000 0.00000100
B -1.85147200 0.00002300 1.44055800
B -0.94692700 1.43966400 0.89870100
C 0.38927900 0.83909200 0.00000700
H -3.37470400 1.54439200 0.00000700
H 0.67002500 -0.00004000 -2.35384500
H 0.67004000 -0.00002900 2.35384600
H -2.43353800 0.00004800 2.46954300
H -0.79394400 2.45148900 1.48727100
H -0.79402500 -2.45148600 1.48729900
H -0.79404400 -2.45151400 -1.48725800
H -2.43346100 0.00001500 -2.46959600
H -0.79397600 2.45150500 -1.48727800
H -3.37477000 -1.54428800 0.00001500
H 2.04777600 -2.38908500 0.00006900

H 2.04774000 2.38909200 0.00027900
H 4.16623600 0.00010000 -0.00005300
12
E(B3LYP/6-311+G**) = -447.578215248
C 2.02828200 -1.20965000 0.04229000
C 2.85047000 -0.00002200 -0.00251800
C 2.02832100 1.20959700 0.04266000
C 0.63246100 0.85127400 0.00704000
C 0.63246700 -0.85109100 0.00676600
B -0.70912400 1.44776900 0.89837400
B -1.60236800 -0.00018100 1.43386200
B -2.13210100 -0.90669500 -0.00779600
B -1.59372100 0.00010200 -1.44198100
B -2.13210500 0.90662400 -0.00755700
B -0.69751600 1.44669300 -0.90071200
B 0.15511000 0.00037200 -1.46565900
B -0.69736600 -1.44635300 -0.90105800
B -0.70915000 -1.44814200 0.89801300
B 0.14685700 -0.00031800 1.46836300
H -3.12933100 1.54096300 -0.01237600
H 0.93446700 0.00051100 -2.34946700
H 0.91703200 -0.00036600 2.36122600
H -2.17304700 -0.00032900 2.46909600
H -0.56519000 2.45589900 1.49525200
H -0.56490700 -2.45663000 1.49420900
H -0.54831700 -2.45439300 -1.49715600
H -2.15681500 0.00024100 -2.48148500
H -0.54855300 2.45512200 -1.49617100
H -3.12936800 -1.54096700 -0.01275100
H 2.40256500 -2.22044900 0.02283000
H 2.40276800 2.22034100 0.02304600
H 3.21775000 0.00022700 -1.05905500
H 3.76635500 -0.00017600 0.59613400

Benzene condensed compounds

Indole (6)
E(B3LYP/6-311+G**) = -363.913933402
C -2.25234700 0.79551100 0.00000000
N -1.09659700 1.55468200 0.00000000
C -0.00000000 0.71598100 0.00000000
C -0.49222100 -0.61770500 0.00000000
C -1.92680000 -0.53316100 0.00000000
H -3.22001700 1.27220500 0.00000000
H -2.62645300 -1.35426300 0.00000000
C 0.42848100 -1.67825200 -0.00000000
C 1.36626700 1.00697400 -0.00000000
H -1.06314500 2.55997900 0.00000000
C 2.25029900 -0.06368200 -0.00000000
H 1.72723300 2.02978800 -0.00000000
H 3.31732400 0.12766000 -0.00000000
C 1.78558500 -1.39353800 -0.00000000
H 0.08133400 -2.70579000 -0.00000000
H 2.50432800 -2.20512700 -0.00000000
Isoindole (6’)
E(B3LYP/6-311+G**) = -363.899396361
C -1.12489100 1.58874100 0.00000000
N -2.21958300 0.76715500 0.00000000
C -1.86636900 -0.55526500 0.00000000
C -0.47375800 -0.60383000 0.00000000
C 0.00000000 0.76643700 -0.00000000
H -1.22582100 2.66127000 -0.00000000
C 1.39631300 1.03000400 -0.00000000
C 0.46302700 -1.67222100 0.00000000
H -2.60860700 -1.33604300 0.00000000
H -3.17195800 1.09642100 0.00000000
C 2.26900400 -0.02777500 -0.00000000
H 1.76212400 2.05124800 -0.00000000
H 3.33768500 0.15614400 -0.00000000
C 1.80215100 -1.37840700 -0.00000000
H 2.52886200 -2.18322700 -0.00000000
H 0.12194000 -2.70200500 0.00000000
Benzotiophene
E(B3LYP/6-311+G**) = -706.757690625
C -2.28108500 -0.46139600 0.00000000
S -1.64588500 1.16874300 0.00000000
C 0.00000000 0.55665400 0.00000000
C 0.01569900 -0.85854900 -0.00000000
C -1.31381400 -1.41122200 0.00000000
H -3.35118600 -0.60586500 0.00000000
H -1.52150200 -2.47348100 0.00000000
C 1.25600100 -1.52075700 -0.00000000
C 1.18087200 1.30340100 -0.00000000
C 2.39111500 0.62371300 -0.00000000
H 1.15508700 2.38683700 -0.00000000
H 3.31852600 1.18487900 -0.00000000
C 2.42778100 -0.78143700 -0.00000000
H 3.38515000 -1.28981600 -0.00000000
H 1.28868300 -2.60487700 -0.00000000
2-Benzotiophene
E(B3LYP/6-311+G**) = -706.740134278
C -1.32652500 1.12763900 -0.00000000
S -2.38302100 -0.22455000 0.00000000
C -1.09253600 -1.35555700 0.00000000
C 0.13620100 -0.71631400 0.00000000
C -0.00000000 0.72911400 -0.00000000
H -1.72578800 2.13002000 -0.00000000
C 1.17592100 1.54221700 -0.00000000
C 1.44330600 -1.29543000 0.00000000
H -1.29762600 -2.41487700 0.00000000
C 2.40490000 0.94597900 -0.00000000
H 1.08145000 2.62245100 -0.00000000
H 3.30116900 1.55616600 -0.00000000
C 2.53926900 -0.48007700 -0.00000000
H 3.53371900 -0.91208100 0.00000000
H 1.55219700 -2.37430000 0.00000000
Benzofuran
E(B3LYP/6-311+G**) = -383.773830443
C -2.18520200 0.86772600 -0.00000000
O -1.02678500 1.60349400 -0.00000000
C 0.00000000 0.69746200 0.00000000
C -0.50966600 -0.61239200 -0.00000000

C -1.94577000 -0.46405500 -0.00000000
H -3.09506400 1.44489500 -0.00000000
H -2.69109700 -1.24367600 -0.00000000
C 0.38973000 -1.68730200 -0.00000000
C 1.35442800 0.99628700 0.00000000
C 2.22847200 -0.08781700 0.00000000
H 1.70693200 2.02007700 0.00000000
H 3.29717500 0.09244600 0.00000000
C 1.75148800 -1.41035600 0.00000000
H 2.46287600 -2.22823900 0.00000000
H 0.03256900 -2.71077600 -0.00000000
2-Benzofuran
E(B3LYP/6-311+G**) = -383.750923904
C 1.75821500 -1.40724800 0.00000000
C 0.42086200 -1.68340800 0.00000000
C -0.49905200 -0.59179200 -0.00000000
C 0.00000000 0.77354000 0.00000000
C 1.40679500 1.01558100 0.00000000
C 2.25143800 -0.05740400 0.00000000
C -1.86964100 -0.49220500 -0.00000000
O -2.23169500 0.81557700 -0.00000000
C -1.11156300 1.58140400 -0.00000000
H -2.67421000 -1.20709500 -0.00000000
H -1.26519500 2.64667100 -0.00000000
H 1.79044100 2.02946900 0.00000000
H 3.32387500 0.10178100 0.00000000
H 2.47544400 -2.22031100 0.00000000
H 0.06087200 -2.70594900 -0.00000000
Benzoborole
E(B3LYP/6-311+G**) = -333.974181826
C -1.71332200 -1.06649000 0.00030400
C -0.28734900 -0.63494000 0.00022200
C -0.22536800 0.78355700 0.00018000
B -1.71338100 1.29206200 -0.00037900
C -2.56880200 -0.01787200 -0.00011400
H -2.09309700 2.41935500 -0.00103900
C 1.01047500 1.40538400 0.00024300
C 0.85671200 -1.40620700 -0.00019100
H -1.99271300 -2.11691600 0.00044400
H -3.64473900 -0.14406500 0.00000500
C 2.10999100 -0.75507500 -0.00024300
H 0.80765100 -2.49035400 -0.00044900
H 3.01863600 -1.34718400 -0.00045800
C 2.18913500 0.62776400 0.00009100
H 3.15838700 1.11362400 0.00006800
H 1.08394600 2.48850200 0.00037000
2-Benzoborole
E(B3LYP/6-311+G**) = -333.924978528
C -1.61453800 -1.25498800 -0.00028600
C -0.35254000 -0.76426300 -0.00003400
C -0.35256200 0.76424400 0.00010100
C -1.61451000 1.25500300 0.00008300
B -2.57635800 -0.00000300 0.00013400
H -1.84110900 2.31416800 0.00027700
C 0.92700000 1.44980700 -0.00014200
C 0.92700900 -1.44981400 0.00004100
H -1.84110600 -2.31416400 -0.00037500
H -3.76592800 0.00002400 0.00045100
C 2.07167600 -0.73384000 0.00012300
H 0.94413600 -2.53435200 0.00015100
H 3.03106200 -1.23968100 0.00045100
C 2.07172400 0.73385300 -0.00006600
H 3.03112200 1.23964700 0.00013500
H 0.94406300 2.53435400 -0.00067100
Quinoline (Benzopyridine)
E(B3LYP/6-311+G**) = -402.031054092
C 0.00000000 -0.70496000 -0.00000000
N -1.12883500 -1.47098300 0.00000000
C -2.29080400 -0.85584500 0.00000000
C -2.44492100 0.55226200 0.00000000
C -1.32070700 1.33884300 0.00000000
C -0.04385600 0.72506300 0.00000000
H -3.17327600 -1.49156800 0.00000000
H -3.43823900 0.98514900 0.00000000
H -1.39385700 2.42181500 0.00000000
C 1.17210500 1.45542200 -0.00000000
C 1.26172400 -1.35281400 -0.00000000
C 2.37929900 0.79822600 -0.00000000
H 1.13363700 2.54007000 -0.00000000
H 3.30505900 1.36221900 -0.00000000
C 2.42263900 -0.61691000 -0.00000000
H 3.38303800 -1.12028300 -0.00000000
H 1.27261300 -2.43623900 -0.00000000
Isoquinoline (2-Benzopyridine)
E(B3LYP/6-311+G**) = -402.029158329
C 2.40412500 0.73968000 0.00000000
C 1.21451500 1.42858700 0.00000000
C -0.01893600 0.72700400 0.00000000
C -0.00000000 -0.69860900 -0.00000000
C 1.24180200 -1.38258800 -0.00000000
C 2.42082500 -0.67659600 0.00000000
C -1.25030100 -1.37422700 -0.00000000
N -2.41848400 -0.77302900 -0.00000000
C -2.42519900 0.58806900 -0.00000000
C -1.28840900 1.35806500 0.00000000
H -1.35822000 2.44036700 0.00000000
H -3.40755600 1.04956000 -0.00000000
H -1.26030600 -2.46312300 -0.00000000
H 1.24652800 -2.46758600 -0.00000000
H 3.37025700 -1.19965300 -0.00000000
H 3.34305700 1.28203100 0.00000000
H 1.20509500 2.51329200 0.00000000
Naphtalene
E(B3LYP/6-311+G**) = -385.988889183
C 2.43051000 -0.70714700 0.00000000
C 1.24381500 -1.40058300 -0.00000000
C 0.00022400 -0.71574900 -0.00000000
C -0.00000000 0.71562300 0.00000000
C 1.24337700 1.40101000 0.00000000
C 2.43031100 0.70796900 0.00000000

C -1.24367000 -1.40103000 -0.00000000
C -1.24392800 1.40054100 0.00000000
H 1.24204100 2.48622100 0.00000000
H 3.37276400 1.24399100 0.00000000
H 3.37312700 -1.24290900 0.00000000
H 1.24276600 -2.48578600 -0.00000000
C -2.43034500 0.70739700 -0.00000000
H -1.24250000 2.48580400 0.00000000
H -3.37323200 1.24263700 -0.00000000
C -2.43032100 -0.70803100 -0.00000000
H -1.24178000 -2.48626000 -0.00000000
H -3.37303100 -1.24370700 -0.00000000
Benzophosphole
E(B3LYP/6-311+G**) = -650.504537835
C -1.05464400 -1.42882800 0.00593300
C 0.04431600 -0.57420000 0.01279000
C -0.14105900 0.82762000 0.00021100
C -1.43594200 1.35741000 -0.01259400
C -2.52932200 0.49568000 0.00188200
C -2.34188900 -0.88958600 0.01502400
H -3.20173400 -1.54994000 0.02394300
H -3.53481100 0.90132700 -0.00177200
P 1.83223000 -0.97792500 -0.11562000
C 2.22567000 0.80044300 0.04336400
C 1.11766800 1.56796800 0.01205100
H -1.58510200 2.43194800 -0.03008100
H -0.91973900 -2.50523000 -0.00520300
H 2.07447900 -1.38558700 1.22872100
H 1.13700400 2.65310900 -0.00042600
H 3.23767100 1.18419800 0.04716100
2-Benzophosphole
E(B3LYP/6-311+G**) = -650.475446950
C 1.33352500 1.42884000 -0.00307700
C 0.06956700 0.73842100 0.02837200
C 0.06953900 -0.73835000 0.02833100
C 1.33348700 -1.42885600 -0.00297800
C 2.49395500 -0.72159900 -0.01265200
C 2.49396400 0.72155700 -0.01290900
H 3.44585400 1.24083700 -0.02624200
H 3.44584000 -1.24089400 -0.02598600
C -1.18663100 1.29032800 0.07392100
P -2.41149000 -0.00000100 -0.14415500
C -1.18672300 -1.29033200 0.07353600
H 1.34155100 -2.51332900 -0.01037900
H 1.34164400 2.51331400 -0.01042800
H -1.39795500 2.35093600 0.05981400
H -1.39780900 -2.35099700 0.06019300
H -3.13086700 0.00010000 1.08008500
Benzosilacyclopentadienyl anion
E(B3LYP/6-311+G**) = -598.668489104
C 2.36099200 -0.88790700 0.03273800
C 1.07509400 -1.43239800 0.03497600
C -0.06733500 -0.62150800 0.03127100
C 0.12290600 0.79762000 0.00703300
C 1.41920400 1.33920800 -0.04006800
C 2.53281600 0.50370400 -0.01540800
C -1.11434300 1.56385900 0.03445800
C -2.26654100 0.83459000 0.09521000
Si -1.93074300 -1.01465000 -0.16548000
H -2.31813500 -1.66496000 1.15862200
H -1.07689300 2.65404800 -0.01031300
H -3.23037600 1.34181900 0.10934400
H 1.55176500 2.41884600 -0.08231500
H 3.53322700 0.92789600 -0.03545300
H 3.23045300 -1.54015300 0.05932100
H 0.96360300 -2.51541500 0.03625300
2-Benzosilacyclopentadienyl anion
E(B3LYP/6-311+G**) = -598.656479331
C 2.48755700 0.70642900 0.00011300
C 1.28718500 1.40101000 0.00009000
C 0.03716500 0.73472400 -0.00026200
C 0.03714600 -0.73469600 -0.00019400
C 1.28714000 -1.40102600 0.00003700
C 2.48753800 -0.70648600 0.00004600
C -1.24922100 -1.35284200 -0.00037900
Si -2.39903000 -0.00002300 0.00087200
C -1.24917900 1.35293500 -0.00071600
H -1.36419000 2.42956600 -0.00024700
H -1.36430600 -2.42946100 -0.00111000
H -3.88475100 0.00001800 -0.00378800
H 1.29413900 -2.48946900 0.00011800
H 3.42964400 -1.24892000 0.00007900
H 3.42968200 1.24883400 0.00018600
H 1.29422400 2.48945300 0.00014600
Triplet state compounds
1-t
E(UB3LYP/6-311+G**) = -463.613030070
C 2.85633400 0.00968300 -0.21549800
N 2.04084200 -1.10841300 0.03704600
C 0.65393800 -0.75878900 0.02903100
C 0.60438100 0.87055600 -0.02542700
C 2.02775800 1.27798600 -0.01887200
B -0.61879700 -1.41212000 0.94100800
B -2.07671800 -0.95601100 0.02513900
B -2.15291200 0.83312200 -0.03997700
B -1.57346300 -0.08826100 -1.45435300
B -0.61928000 -1.47925800 -0.84926000
B 0.16771200 -0.02375100 -1.47490800
B -0.73162100 1.41028700 -0.94811700
B -0.74824400 1.47880700 0.83677300
B 0.14669000 0.09628800 1.47144400
B -1.58631500 0.01825200 1.44590900
H -3.04255000 -1.63597300 0.05021300
H 0.94393200 0.14025800 2.33660300
H 0.96876200 -0.03432600 -2.33343100
H -2.16399400 -0.14755300 -2.47564100
H -0.41364000 -2.50518500 -1.39209400
H -0.61276700 2.40250700 -1.57281900
H -0.63316300 2.51642600 1.38591300
H -2.18720700 0.02559000 2.46315300

H -0.41467300 -2.39395200 1.56412700
H -3.17367900 1.42724400 -0.06673900
H 3.89946800 -0.06104400 0.05793600
H 2.32037100 -1.83456600 0.68743900
H 2.33351300 2.07607200 0.65232600
1’-t
E(UB3LYP/6-311+G**) = -463.636534493
B 2.12956300 0.89643500 -0.02899000
B 0.71042900 1.44480600 0.89445400
C -0.62414000 0.80813300 0.02864300
C -0.62422900 -0.80828400 0.02849900
C -2.05222100 -1.17598000 0.06499300
N -2.84434100 0.00005100 -0.07090200
C -2.05214500 1.17603500 0.06500200
B 2.12950200 -0.89644400 -0.02897800
B 1.56942600 0.00004300 -1.47040300
B 0.67381400 1.44449400 -0.89320000
B -0.16534300 -0.00002800 -1.47038700
B 0.67390300 -1.44452800 -0.89326200
B 0.71029400 -1.44476000 0.89439500
B -0.11123200 0.00008100 1.49869900
B 1.62729300 -0.00000900 1.43116100
H 3.12267200 1.53653600 -0.04928800
H -0.88293800 -0.00009300 2.38501900
H -0.97892400 0.00006300 -2.31927000
H 2.14940300 0.00007200 -2.49997500
H 0.50227500 2.45825100 -1.47171600
H 0.50244000 -2.45834100 -1.47170000
H 0.56516500 -2.45783100 1.48034800
H 2.24376000 -0.00020300 2.43921000
H 0.56489100 2.45788200 1.48029800
H 3.12261300 -1.53654300 -0.04929500
H -3.75910200 0.00006900 0.36329500
H -2.43169400 2.12368000 -0.29077100
H -2.43201800 -2.12377900 -0.29010500
2-t
E(UB3LYP/6-311+G**) = -806.470828915
C 2.70705500 0.70648500 -0.23320700
S 2.31197500 -0.95860800 0.09873900
C 0.49790400 -0.64238500 0.04303300
C 0.28133000 0.99051600 0.03528000
C 1.61304500 1.62750200 0.14423300
B -0.73387000 -1.46105900 0.88438100
B -2.19696100 -1.11590100 -0.06696900
B -2.45586400 0.65408600 -0.07042400
B -1.73224100 -0.14451300 -1.49588300
B -0.65787500 -1.45010600 -0.90261800
B -0.00547600 0.10895400 -1.44287600
B -1.08443700 1.41376100 -0.91030000
B -1.14454900 1.40418600 0.87431500
B -0.12277900 0.08968000 1.48906800
B -1.84805100 -0.15444300 1.40260500
H -3.08606200 -1.89289700 -0.10603100
H 0.62061000 0.19872400 2.39306500
H 0.80471500 0.21935200 -2.28681400
H -2.27340200 -0.23014500 -2.54248400
H -0.33154500 -2.42193300 -1.48441400
H -1.04868000 2.43660500 -1.49493400
H -1.15332100 2.42266100 1.46975300
H -2.47226900 -0.24173000 2.40164600
H -0.44784100 -2.43717300 1.48058900
H -3.53158600 1.14091000 -0.10797800
H 3.49784600 0.92501900 -0.93883300
H 1.74445000 2.40241500 0.89407900
2’-t
E(UB3LYP/6-311+G**) = -806.490040103
S 2.96394200 -0.00007700 -0.03828500
C 1.74903100 -1.28794800 0.01996800
C 0.35966500 -0.81034600 0.00640200
C 0.35948300 0.81054100 0.00637800
C 1.74875000 1.28848400 0.01952800
B -0.97087300 -1.44297900 0.88937200
B -2.40125600 -0.89518000 -0.01305100
B -2.40143500 0.89485400 -0.01329800
B -1.85531400 -0.00027400 -1.46022900
B -0.95801100 -1.44306500 -0.89689600
B -0.11303000 -0.00013500 -1.47944900
B -0.95828700 1.44285300 -0.89721600
B -0.97114600 1.44310300 0.88907500
B -0.13679600 0.00023100 1.48609200
B -1.87586400 0.00005300 1.44254000
H -3.39311000 -1.53703700 -0.01977500
H 0.64872600 0.00027600 2.36074100
H 0.68385800 0.00005100 -2.34263100
H -2.44183300 -0.00035700 -2.48569200
H -0.79190400 -2.45592800 -1.47710600
H -0.79241000 2.45566900 -1.47756100
H -0.81163600 2.45632100 1.47163500
H -2.47823300 0.00005600 2.45886900
H -0.81098700 -2.45606900 1.47204900
H -3.39343000 1.53649200 -0.02003900
H 2.03328900 -2.28742600 0.31131100
H 2.03308000 2.28750100 0.31241200
3-t
E(UB3LYP/6-311+G**) = -483.471058470
C 2.80379600 -0.05560700 -0.22626200
O 1.99548500 -1.12703000 0.15644500
C 0.65718700 -0.74605100 0.08090400
C 0.60935100 0.87874100 -0.05506900
C 2.03705100 1.25142200 -0.03269000
B -0.61516900 -1.37010400 1.00979000
B -2.05791100 -0.95741600 0.05055300
B -2.14073700 0.82710900 -0.09639500
B -1.53893700 -0.15568500 -1.46258700
B -0.58417200 -1.51656000 -0.77989000
B 0.19610400 -0.07887200 -1.46620300
B -0.71879100 1.37324200 -1.01724200
B -0.74561400 1.51741700 0.76666800
B 0.14424700 0.16187900 1.47651600
B -1.59037100 0.08286600 1.43018700

H -3.02192100 -1.63857300 0.09152300
H 0.93659300 0.24769800 2.34186600
H 1.01531300 -0.13103500 -2.30709900
H -2.11823400 -0.26771000 -2.48559500
H -0.36602000 -2.56310500 -1.27436100
H -0.59397400 2.33817300 -1.68191200
H -0.63974300 2.57826800 1.27093200
H -2.20332500 0.13952700 2.43818300
H -0.41176500 -2.31983900 1.67648600
H -3.16366500 1.41398800 -0.16076800
H 3.84394800 -0.20037700 0.03255400
H 2.37136200 2.02881400 0.64836000
3’t
E(UB3LYP/6-311+G**) = -483.494158669
O 2.85238300 0.00006500 0.01623600
C 2.06196400 -1.14749500 -0.08395600
C 0.62831000 -0.80407200 -0.03987600
C 0.62820600 0.80388700 -0.04030900
C 2.06186300 1.14764900 -0.08406500
B -0.65738500 -1.44532600 0.89756800
B -2.11931600 -0.89696800 0.04585500
B -2.11944100 0.89694100 0.04561200
B -1.63348100 -0.00023900 -1.42024900
B -0.70904100 -1.44598400 -0.89281000
B 0.10193600 -0.00010700 -1.51172500
B -0.70931900 1.44578600 -0.89331400
B -0.65735900 1.44553900 0.89701900
B 0.18835500 0.00016600 1.46594100
B -1.54671200 0.00025100 1.48250200
H -3.11193600 -1.53665100 0.07576200
H 1.01548900 0.00049700 2.30239100
H 0.87161600 -0.00047100 -2.39925400
H -2.26156500 -0.00067800 -2.42051200
H -0.56787000 -2.45961600 -1.47779900
H -0.56752100 2.45921900 -1.47850000
H -0.47604900 2.45952800 1.47139700
H -2.11474200 0.00041700 2.51830700
H -0.47621700 -2.45921500 1.47216200
H -3.11210400 1.53656200 0.07529600
H 2.50456900 -2.04618900 0.31842700
H 2.50402900 2.04598500 0.31966300
4-t
E(UB3LYP/6-311+G**) = -433.729307738
C -2.08150100 -1.19551600 0.00925800
C -2.96439900 0.02534900 0.01882300
B -2.15668800 1.32059200 -0.02929800
C -0.63893000 0.89383800 -0.01925700
B -0.21427500 0.07113200 1.44686000
B 0.70215700 1.47401000 0.85510200
B 0.70515900 1.43574300 -0.91843800
B -0.21606200 0.00841200 -1.44586800
B 0.61831900 -1.46559400 -0.85766600
B 2.06561700 -0.93830000 0.02210200
B 1.54314600 -0.05523500 -1.44749400
B 2.12243800 0.85544200 -0.01913200
B 1.54439200 0.01067400 1.44966600
B 0.62079400 -1.42707700 0.92669700
C -0.67780500 -0.80361400 0.02146000
H 3.04335500 -1.60195300 0.03591500
H -0.98177800 0.00052600 -2.34341200
H -0.98024300 0.10427100 2.34307000
H 2.12378200 0.01519300 2.47921800
H 0.43306400 -2.41159300 1.54893900
H 0.58633800 2.49281600 1.43794400
H 0.58756600 2.42723400 -1.54646600
H 2.11892800 -0.09852700 -2.47823700
H 0.42955900 -2.47697500 -1.43559600
H 3.14085300 1.45441200 -0.03325600
H -2.55604200 2.43345200 -0.03807400
H -2.40552900 -2.21085200 -0.16690300
H -4.03902600 -0.09733400 0.10249200
4’-t
E(UB3LYP/6-311+G**) = -433.745916104
C 2.09135000 1.25070700 -0.00024500
B 3.01583800 -0.00004300 -0.00001300
C 2.09127500 -1.25072900 0.00016900
C 0.67847500 -0.84126900 0.00005300
B 0.22068000 0.00050200 1.45566500
B -0.64641500 -1.45038200 0.89055600
B -0.64652500 -1.45112100 -0.88955000
B 0.22052600 -0.00051200 -1.45571000
B -0.64644500 1.45043700 -0.89056300
B -2.07965500 0.89655900 -0.00026100
B -1.53193500 -0.00048500 -1.44806700
B -2.07968800 -0.89650700 0.00041000
B -1.53179100 0.00049600 1.44813600
B -0.64640600 1.45106000 0.88955600
C 0.67851400 0.84126600 -0.00025800
H -3.07657700 1.53050100 -0.00046700
H 0.99362600 -0.00094500 -2.34400200
H 0.99384500 0.00083200 2.34389500
H -2.11097000 0.00086900 2.47774500
H -0.49022800 2.45561100 1.48779500
H -0.49024300 -2.45451200 1.48950600
H -0.49049000 -2.45575400 -1.48768100
H -2.11123000 -0.00084600 -2.47760900
H -0.49033300 2.45465000 -1.48938500
H -3.07665300 -1.53038400 0.00070800
H 2.33508900 -2.30564500 0.00016500
H 2.33491500 2.30568300 0.00026000
H 4.20064300 0.00006700 -0.00003800
5-t
E(UB3LYP/6-311+G**) = -501.722883352
B -1.79552500 0.01543600 -1.44786300
B -2.33656500 -0.88303800 0.00000600
B -2.31993200 0.91048800 0.00001400
B -1.79550700 0.01542900 1.44787600
B -0.04720200 -0.00877900 1.47877300
C 0.41278600 -0.84576300 -0.00001300
N 1.67851000 -1.37198500 -0.00002900

C 2.89127900 -0.79461900 0.00004300
C 2.94331800 0.61080500 -0.00001700
C 1.81593800 1.40969700 -0.00000500
C 0.45386800 0.83327800 -0.00000100
B -0.04721900 -0.00877100 -1.47878100
B -0.91467800 -1.43909500 -0.90519100
B -0.91466400 -1.43910200 0.90517900
B -0.87315800 1.43737200 0.89587000
B -0.87316800 1.43737700 -0.89585500
H -3.30074500 1.56893900 0.00002000
H 0.74424100 -0.02794600 -2.34725100
H 0.74426800 -0.02796000 2.34723500
H -2.38824600 0.02545200 2.46971800
H -0.70327000 2.44372800 1.48750400
H -0.76023500 -2.45467500 1.48328000
H -0.76026000 -2.45466400 -1.48330300
H -2.38827800 0.02547100 -2.46969700
H -0.70328000 2.44373400 -1.48748700
H -3.33027900 -1.52210800 0.00001200
H 3.92994400 1.05838300 -0.00006100
H 3.76566100 -1.43002800 0.00004400
H 1.88586200 2.48860000 0.00001100

5’-t

E(UB3LYP/6-311+G**) = -501.750527489

C 0.42678100 0.81761400 0.11098700
C 0.43705000 -0.81619100 0.01109700
C 1.78246000 -1.41772800 0.11067500
N 2.93917600 -0.69832600 -0.12172700
C 2.94038300 0.59187400 -0.05812400
C 1.76477500 1.42188300 0.18372200
B -0.12018600 -0.07305100 1.50467600
B -0.95271100 1.39648500 0.95515600
B -0.84947700 1.48657200 -0.81959600
B 0.03996100 0.07381200 -1.43348900
B -0.84672700 -1.39108900 -0.98023900
B -0.93068800 -1.48282200 0.79551600
B -1.86302200 -0.07449500 1.38884700
B -2.33439500 0.89282700 -0.04155800
B -2.32727200 -0.88927500 -0.13445400
B -1.70847100 0.08117100 -1.50630500
H -3.32584700 1.53495400 -0.06065900
H 0.87116700 0.12765100 -2.26290800
H 0.61738500 -0.12145100 2.41892700
H -2.50706100 -0.13427600 2.37724900
H -0.82019800 2.37627700 1.59820700
H -0.79362800 -2.52303200 1.33462100
H -0.64871300 -2.36959600 -1.60691300
H -2.24080100 0.14283400 -2.55904700
H -0.65523400 2.52786000 -1.33851000
H -3.31464600 -1.53242800 -0.21886000
H 3.87542900 1.10166300 -0.27913700
H 1.84250500 2.50079100 0.17537100
H 1.88165200 -2.42835700 0.48083900

I-t

E(UB3LYP/6-311+G**) = -485.713990554

B -2.34683300 0.89100400 0.00001100
B -1.79942400 0.00001000 -1.45118400
B -0.90927200 -1.43886200 -0.88866900
C 0.42742100 -0.81870500 -0.00001000
C 1.74853000 -1.46589000 -0.00001000
C 2.96849500 -0.67836900 0.00000200
C 2.96849600 0.67836900 0.00000700
C 1.74853000 1.46589100 -0.00000500
C 0.42742000 0.81870500 0.00000300
B -0.90926600 1.43886200 0.88867200
B -1.79941600 -0.00001000 1.45118900
B -0.04914100 -0.00000900 1.47098600
B -0.90926400 -1.43887400 0.88865100
B -2.34683300 -0.89100500 -0.00000200
B -0.04914900 0.00001000 -1.47098800
B -0.90927000 1.43887300 -0.88864800
H -0.74410300 -2.45017400 1.47288500
H -3.33630300 1.53726500 0.00001800
H -3.33630400 -1.53726400 -0.00000000
H -2.38857700 -0.00001800 2.47542700
H -0.74411100 2.45017300 -1.47288500
H -0.74410300 2.45015400 1.47292100
H -2.38859300 0.00001700 -2.47541700
H -0.74411000 -2.45015300 -1.47292000
H 0.73093600 -0.00001500 2.35021900
H 0.73092000 0.00001400 -2.35022800
H 1.77586800 -2.54608400 -0.00000900
H 3.90930100 -1.21562400 0.00001100
H 1.77586900 2.54608500 -0.00004800
H 3.90930100 1.21562400 0.00002000

8-t

E(UB3LYP/6-311+G**) = -639.417443171

C -4.39871800 0.69191200 0.00000100
C -3.17842000 1.39069400 0.00000000
C -1.94866600 0.72239200 -0.00000100
C -1.94866600 -0.72239200 0.00000200
C -3.17842000 -1.39069400 -0.00000000
C -4.39871800 -0.69191200 -0.00000200
C -0.72757600 1.45713800 -0.00000000
C 0.59470300 0.81373500 -0.00000000
B 1.92921900 1.43680100 -0.89025700
B 2.82437700 -0.00000100 -1.45031300
B 3.36696500 0.89332000 -0.00000100
B 2.82437800 0.00000100 1.45031200
B 1.92921900 -1.43680100 0.89025700
B 1.07435900 0.00000100 1.47448600
B 1.92921900 1.43680200 0.89025500
C -0.72757700 -1.45713800 0.00000200
C 0.59470300 -0.81373500 0.00000100
B 1.92921800 -1.43680200 -0.89025500
B 3.36696500 -0.89332000 -0.00000000
B 1.07435700 -0.00000100 -1.47448600
H 1.76490000 2.45057600 1.47099800
H 4.35661000 -1.53930400 0.00000000
H 4.35661000 1.53930400 -0.00000200

H 3.41315300 0.00000100 2.47486100
H 1.76489900 -2.45057600 -1.47099700
H 1.76490100 -2.45057300 1.47100200
H 3.41315100 -0.00000200 -2.47486200
H 1.76489900 2.45057400 -1.47100200
H 0.29111700 0.00000200 2.35132800
H 0.29111400 -0.00000200 -2.35132700
H -0.74417000 2.53910300 -0.00000000
H -0.74417000 -2.53910300 0.00000100
H -3.18321400 -2.47539100 -0.00000200
H -5.33192200 -1.24239700 -0.00000300
H -3.18321400 2.47539100 0.00000200
H -5.33192200 1.24239700 0.00000200
9-t
E(UB3LYP/6-311+G**) = -617.343346395
C -3.47796700 1.12934100 -0.00005400
C -2.14605200 0.73084900 0.00001100
C -2.14605200 -0.73084900 -0.00002600
C -3.47796700 -1.12934100 0.00004300
N -4.27400700 0.00000000 0.00002400
C -0.96650500 1.49495700 0.00004000
C 0.34988400 0.82463700 0.00002400
B 0.82784800 0.00004500 -1.46708800
B 1.68692700 -1.43522200 -0.89055800
B 3.12488900 -0.89346700 -0.00002400
B 1.68692400 -1.43527700 0.89047300
B 0.82784300 -0.00004500 1.46708600
B 1.68692400 1.43522200 0.89056000
B 2.58129900 -0.00004400 1.44860800
B 3.12488900 0.89346700 0.00003100
B 2.58130400 0.00004400 -1.44860300
B 1.68692700 1.43527700 -0.89047100
C -0.96650500 -1.49495700 -0.00004800
C 0.34988400 -0.82463700 -0.00002600
H -0.97375600 2.57609800 0.00003900
H -0.97375600 -2.57609800 -0.00004100
H -3.90866800 2.11772600 -0.00009000
H -5.27973900 0.00000000 -0.00002200
H 1.52621900 2.44816300 1.47466500
H 4.11471400 -1.54009800 -0.00004200
H 4.11471400 1.54009800 0.00005200
H 3.16913200 -0.00007500 2.47421400
H 1.52622500 -2.44816300 -1.47466400
H 1.52622100 -2.44825300 1.47451600
H 3.16914100 0.00007500 -2.47420700
H 1.52622600 2.44825300 -1.47451500
H 0.04942700 -0.00007200 2.34862800
H 0.04943500 0.00007200 -2.34863100
H -3.90866800 -2.11772600 0.00008400
10-t
E(UB3LYP/6-311+G**) = -739.225638082
C -1.30108000 -0.81551200 -0.00015000
B -2.64642600 -1.43756000 0.89004600
B -3.54445400 0.00004300 1.44670200
B -4.08464900 0.89439000 0.00017500
B -2.64663500 1.43758000 -0.89002200
B -2.64637400 1.43759600 0.89003800
C -1.30119500 0.81547600 -0.00006200
B -1.79971700 -0.00001400 -1.47897500
B -3.54473900 -0.00004900 -1.44653000
B -4.08461700 -0.89441600 0.00011000
B -2.64648800 -1.43759400 -0.89003100
B -1.79940500 0.00007400 1.47882100
C 0.00003700 -1.50333600 0.00002100
C 1.30114900 -0.81551200 -0.00013600
B 2.64652400 -1.43758300 -0.89003200
B 1.79970900 -0.00004100 -1.47897200
B 2.64660300 1.43757800 -0.89002700
B 2.64633800 1.43760700 0.89003700
B 3.54446000 0.00006900 1.44670800
B 4.08462600 0.89442600 0.00016600
B 3.54473300 -0.00002200 -1.44652400
B 4.08463900 -0.89438000 0.00010100
B 2.64645800 -1.43756300 0.89004100
B 1.79941300 0.00004600 1.47882400
C -0.00003700 1.50322600 -0.00028100
C 1.30112500 0.81547700 -0.00004800
H -5.07117700 1.54377000 0.00040500
H -2.47903000 -2.44931900 1.47236600
H -1.04651000 0.00023200 -2.37497600
H -2.47935700 -2.44929700 -1.47251600
H -2.47936300 2.44935100 -1.47235500
H -4.12452100 -0.00007400 -2.47548300
H -1.04599700 -0.00013700 2.37467500
H -4.12403400 0.00005400 2.47576900
H -5.07112200 -1.54383000 0.00007400
H -2.47915800 2.44930400 1.47249100
H 4.12404500 0.00009900 2.47577100
H 4.12451000 -0.00002800 -2.47548100
H 5.07113700 1.54383300 0.00039000
H 5.07116200 -1.54376800 0.00005700
H 1.04649800 0.00019400 -2.37496900
H 2.47941500 -2.44928500 -1.47252600
H 2.47909900 2.44931600 1.47248300
H 1.04601000 -0.00017700 2.37468200
H 2.47908800 -2.44932800 1.47235400
H 2.47930500 2.44934300 -1.47236600
H 0.00002100 2.58388600 -0.00065100
H -0.00002200 -2.58400000 0.00047100
11-t
E(UB3LYP/6-311+G**) = -698.457664697
C 1.77529800 1.33135300 -0.05269600
C 0.38780300 0.82977200 -0.01747400
B -0.95183100 1.43536200 0.88854200
B -1.85079200 0.00000000 1.45371700
B -2.40566700 -0.89489400 0.01611400
B -1.88098300 -0.00000000 -1.43430200
B -2.40566700 0.89489400 0.01611400
B -0.97329100 1.43573900 -0.88814500
B -0.12993000 -0.00000000 -1.46687800

B -0.97329100 -1.43573900 -0.88814400
B -0.95183100 -1.43536200 0.88854300
B -0.09938900 0.00000100 1.45031100
C 0.38780300 -0.82977200 -0.01747400
C 1.77529900 -1.33135300 -0.05269600
Si 3.08340000 0.00000000 0.13349000
H -3.40229600 1.53903100 0.02760700
H 0.61353700 0.00000000 -2.37728900
H 0.66407400 0.00000100 2.34345700
H -2.43783600 0.00000000 2.48475600
H -0.80755000 2.45050300 1.47812100
H -0.80755000 -2.45050200 1.47812200
H -0.84363500 -2.45074800 -1.48158100
H -2.48958400 -0.00000100 -2.45288900
H -0.84363600 2.45074800 -1.48158300
H -3.40229500 -1.53903100 0.02760800
H 1.89263700 -2.41017600 -0.06329000
H 1.89263700 2.41017600 -0.06329000
H 3.96003900 -0.00000000 -1.12593300
12-t
E(UB3LYP/6-311+G**) = -750.254096727
C 1.74037600 -1.30237100 -0.00161500
C 0.34993300 -0.81900100 0.00198500
B -0.97497800 -1.44410000 -0.89389400
B -1.86730700 0.00000000 -1.45627400
B -2.41445900 0.89471900 -0.00811400
B -1.88039800 -0.00000100 1.44377700
B -2.41445900 -0.89471900 -0.00811500
B -0.98068900 -1.44366000 0.88906100
B -0.13648600 -0.00000000 1.47521300
B -0.98069000 1.44366000 0.88906200
B -0.97497800 1.44410000 -0.89389300
B -0.12242900 0.00000100 -1.47146600
C 0.34993300 0.81900100 0.00198600
C 1.74037600 1.30237100 -0.00161400
P 2.99515000 -0.00000000 0.09311500
H -3.40730900 -1.53529200 -0.01177500
H 0.64443400 -0.00000100 2.35443000
H 0.66534300 0.00000100 -2.34455600
H -2.45230900 0.00000100 -2.48285400
H -0.81197200 -2.45519400 -1.47913000
H -0.81197200 2.45519500 -1.47912800
H -0.82147100 2.45485800 1.47513400
H -2.47470600 -0.00000100 2.46491100
H -0.82147000 -2.45485900 1.47513200
H -3.40731000 1.53529100 -0.01177400
H 1.95647900 2.36115200 0.03256100
H 1.95647900 -2.36115200 0.03256200
H 3.50920000 0.00000000 -1.25348100
13-t
E(UB3LYP/6-311+G**) = -447.596414479
C 2.94319300 -0.00009200 0.00068600
C 2.03319600 1.20024600 -0.00082100
C 0.61329200 0.81720000 -0.00043800
C 0.61334800 -0.81711300 0.00006100
C 2.03323700 -1.20011100 -0.00027100
B -0.70760500 1.44555200 0.89220600
B 0.13449800 0.00043700 1.47416600
B -0.70755500 -1.44504200 0.89305800
B -0.70778800 -1.44555000 -0.89211800
B 0.13401300 -0.00036900 -1.47439100
B -0.70794100 1.44505300 -0.89298600
B -2.14469400 0.89616300 0.00000700
B -1.60853000 -0.00045900 -1.44975200
B -2.14462700 -0.89624700 0.00051400
B -1.60805500 0.00038700 1.45010400
H -3.13870400 1.53526500 -0.00001300
H 0.92045600 -0.00063900 -2.34975300
H 0.92123000 0.00076000 2.34926800
H -2.19952700 0.00065200 2.47314100
H -0.55051800 2.45736800 1.47814000
H -0.55046700 -2.45652900 1.47955900
H -0.55088900 -2.45738500 -1.47807600
H -2.20033300 -0.00084400 -2.47259800
H -0.55101400 2.45654000 -1.47953300
H -3.13858300 -1.53543600 0.00086400
H 2.36110300 -2.22880100 -0.00270400
H 2.36140400 2.22881800 -0.00136900
H 3.62126600 -0.00038000 -0.87052600
H 3.61840100 0.00020300 0.87425300

ISE compounds (Figure 3)

- 1. reaction:
  - substance: 1s
  - product: 1p
- 2. reaction:
  - substance: 2s
  - product: 2p
- 3. reaction:
  - substance: 3s
  - product: 3p

1_1s
E(B3LYP/6-311+G**) = -503.058029120
C -2.36831700 1.05332300 0.00292400
N -1.24180700 1.89236900 -0.08313800
C -0.06763900 1.08670500 -0.03031300
C -0.60938300 -0.43760500 0.00633300
B 0.43499100 -1.45454200 0.92717000
B 1.75813500 -0.37122500 1.45566500
B 2.55438000 0.28009700 -0.00326300
B 1.76810100 -0.43152400 -1.43523400
B 1.96849200 -1.42356500 0.03119000
B 0.42845300 -1.47977200 -0.86649800
B 0.12620400 0.13938400 -1.48802900
B 1.38385400 1.23999900 -0.93026300
B 1.36463700 1.27826900 0.86981300
B 0.11628400 0.18505200 1.47597700
C -2.09525000 -0.26490800 0.00981200
H 2.70073000 -2.35083600 0.04968700
H -0.62292700 0.38460300 -2.36016300
H -0.63537000 0.47226400 2.33451000

H 2.31708500 -0.53826500 2.48351700
H -0.03491800 -2.35744900 1.52398300
H 1.54014700 2.30312800 1.43023600
H 1.56305300 2.24161800 -1.52703700
H 2.32933700 -0.65022400 -2.45191600
H -0.03974000 -2.40178900 -1.43462400
H 3.70185200 0.56315800 -0.00154500
H -1.23182500 2.75400800 0.44538400
C -3.05243200 -1.41134600 -0.00313000
H -3.34782400 1.51063300 0.01261700
H -2.88843000 -2.07204600 0.85389900
H -4.08322900 -1.05422800 0.03336400
H -2.93481500 -2.01903500 -0.90633600
1_1p
E(B3LYP/6-311+G**) = -503.044047934
C -2.51759700 0.93595700 0.16762100
N -1.35527300 1.82850400 -0.08546500
C -0.15624800 1.05317900 -0.04567800
C -0.57047000 -0.53565600 0.01270200
B 0.53042400 -1.42421200 0.96179000
B 1.76926200 -0.23342500 1.46525100
B 2.51919900 0.42692000 -0.01442200
B 1.77431000 -0.37752000 -1.42915600
B 2.06756200 -1.30613800 0.07200400
B 0.54884400 -1.52672900 -0.82056100
B 0.09085700 0.04990700 -1.46899200
B 1.26093700 1.27767300 -0.94912100
B 1.27909300 1.36640300 0.83719500
B 0.08128000 0.22458400 1.44625800
C -2.06487200 -0.52076300 0.01835700
H 2.86939700 -2.17283000 0.11837600
H -0.67353200 0.22782500 -2.34764400
H -0.68445400 0.45960200 2.30738000
H 2.33177200 -0.34046400 2.49864100
H 0.11509000 -2.32320500 1.60201000
H 1.37683300 2.41251100 1.37063400
H 1.36398000 2.26354800 -1.59038600
H 2.35255600 -0.56838400 -2.44181600
H 0.14179800 -2.49345300 -1.36056200
H 3.64274700 0.79226000 -0.03886700
C -2.84838600 -1.58909700 -0.06978200
H -3.33038800 1.18106400 -0.51769700
H -2.43867200 -2.58944400 -0.13705300
H -3.92835100 -1.49423900 -0.07187600
H -1.43508600 2.29624400 -0.97976000
H -2.88018400 1.10040600 1.18632100
1_2s
E(B3LYP/6-311+G**) = -249.557844215
C -1.46490600 0.71399200 0.00002800
C -0.16003200 1.15238400 -0.00001500
C 0.69070700 0.00359400 0.00000100
C -0.13704200 -1.09853600 0.00001900
N -1.44257800 -0.65859700 -0.00002500
H 0.09818500 -2.15112300 0.00002900
H -2.25429900 -1.25239000 -0.00005200
H -2.39465700 1.25958500 0.00004400
H 0.15521400 2.18515800 -0.00002900
C 2.19141600 -0.00583800 -0.00000100
H 2.57850000 -1.02769000 -0.00000700
H 2.59712500 0.50154000 -0.88129900
H 2.59712600 0.50153000 0.88130200
1_2p
E(B3LYP/6-311+G**) = -249.524075716
C 1.34098000 -0.78319900 0.01187000
C 0.06020300 -1.20478500 -0.05770500
C -0.82015200 -0.04344500 -0.02571100
C 0.10678800 1.17064900 -0.06421400
N 1.45112900 0.59632600 0.15596800
H -0.13443300 1.90808300 0.70414500
H 2.23842700 -1.38779500 0.01997600
H -0.26651300 -2.23354600 -0.07182800
C -2.15923300 -0.00457100 0.04476900
H -2.70295000 0.93253200 0.07867100
H -2.74528000 -0.91559600 0.06923800
H 0.05376800 1.66297900 -1.04312600
H 2.22756200 1.05116500 -0.30290400
1_3s
E(B3LYP/6-311+G**) = -250.750331246
C 1.53573500 -0.62990300 -0.15012000
C 0.12025800 -1.18477000 0.12321400
C -0.75089100 0.05170300 -0.02907100
C 0.04755000 1.12597600 0.00695800
N 1.41256100 0.80933700 0.17852400
H -0.25138500 2.16698900 -0.00770900
H 2.31292100 -1.10394800 0.45165700
H 0.03937400 -1.58593600 1.14391800
C -2.24425300 0.00900100 -0.04856500
H -2.67165400 1.01111500 -0.13616600
H -2.64625400 -0.44619300 0.86619500
H 1.78691800 -0.76287200 -1.21184300
H -0.14563100 -1.99585300 -0.56148200
H 2.05661200 1.40913600 -0.32150000
H -2.61922200 -0.58984100 -0.88723900
1_3p
E(B3LYP/6-311+G**) = -250.739427372
C 1.42356200 -0.66914200 -0.23354700
C 0.04355600 -1.22372800 0.14713200
C -0.86428300 -0.01304800 0.01760900
C 0.02684300 1.21710500 -0.05810000
N 1.35886800 0.70050000 0.29135600
H -0.28234100 2.00241200 0.63692200
H 2.25046200 -1.21871300 0.22178700
H 0.05792400 -1.56450200 1.18775600
C -2.19298700 -0.01796300 -0.04182000
H -2.76381800 0.90124200 -0.12449100
H -2.75935800 -0.94254400 -0.00866300
H -0.01828200 1.63442000 -1.07898800
H 1.54979700 -0.70494700 -1.32952300
H -0.27440600 -2.05965100 -0.47908500
H 2.10780200 1.28943300 -0.05284900

2_1s
E(B3LYP/6-311+G**) = -845.906431391
C -1.87887400 0.81317200 0.00009000
C -2.55570100 -0.34447800 0.00018000
S -1.63895800 -1.85907200 -0.00009900
C -0.06942700 -0.95904000 0.00006500
C -0.39416300 0.63655600 0.00000300
B 0.23077900 -0.07479000 1.47505700
B 1.33259800 -1.33388500 0.89706000
B 1.33234000 -1.33388600 -0.89681400
B 0.23068700 -0.07478200 -1.47508100
B 1.94321700 0.25484700 -1.44700300
B 2.30018300 1.23572400 -0.00008100
B 0.78581600 1.49890000 0.89496600
B 1.94328900 0.25497700 1.44698400
B 2.64076300 -0.52582400 0.00000400
B 0.78578600 1.49884900 -0.89512100
C -2.48678900 2.18303600 -0.00002200
H 3.15373500 2.05291400 -0.00008000
H -0.54214800 -0.22680500 2.34736100
H -0.54210700 -0.22711300 -2.34743900
H 2.51997000 0.36662600 -2.47213100
H 0.44608200 2.46440800 -1.48122100
H 1.36157400 -2.35733100 -1.48167700
H 1.36172300 -2.35739000 1.48181200
H 2.52002900 0.36675400 2.47212000
H 0.44601700 2.46439200 1.48112900
H 3.73604900 -0.96907200 -0.00021500
H -3.63405700 -0.43305700 0.00038200
H -2.17280400 2.75138100 -0.88079300
H -3.57573800 2.12172500 0.00007200
H -2.17256900 2.75159000 0.88051600
2_1p
E(B3LYP/6-311+G**) = -845.895325212
C 2.65915100 -0.17292300 0.34258400
S 1.76046600 -1.71376300 -0.17164200
C 0.14211200 -0.90583400 -0.07228500
C 0.33280200 0.70854800 0.04363900
B -0.90384100 1.40459800 1.00939900
B -1.94947200 0.03518700 1.47085400
B -2.59490700 -0.68815900 -0.03155900
B -1.97293200 0.25493200 -1.41873400
B -2.40059100 1.08757600 0.10320900
B -0.92119800 1.54158500 -0.76914100
B -0.23810400 0.06585400 -1.47144700
B -1.23195000 -1.31977900 -0.98698600
B -1.22472900 -1.45294800 0.79805800
B -0.21078500 -0.16330900 1.45636900
C 1.79507500 1.03917100 0.04729700
H -3.31600700 1.83198200 0.16678900
H 0.53344700 0.04386900 -2.35656700
H 0.57067600 -0.31572100 2.32174200
H -2.52146200 0.02226900 2.50456800
H -0.61855800 2.34005500 1.66825000
H -1.16784700 -2.51558600 1.30442000
H -1.18970600 -2.29439600 -1.64894500
H -2.56864800 0.39683700 -2.42901700
H -0.65815400 2.56899300 -1.28503700
H -3.65138400 -1.21616000 -0.06536700
C 2.27556600 2.25821100 -0.17849200
H 3.60184500 -0.13527900 -0.20212600
H 1.62107500 3.10298900 -0.35259700
H 3.34279100 2.44573700 -0.18772300
H 2.86878700 -0.24610900 1.41132600
2_2s
E(B3LYP/6-311+G**) = -592.401398425
C -1.03660800 1.15241600 0.00000000
C 0.32438600 1.24543100 -0.00000000
C 0.98298900 -0.02880900 -0.00000000
C 0.08187600 -1.05801500 -0.00000000
S -1.56066800 -0.49862200 0.00000000
H 0.28586900 -2.11833500 -0.00000100
H -1.76385600 1.94979800 0.00000000
H 0.85300900 2.19075200 -0.00000100
C 2.47759700 -0.19863800 0.00000000
H 2.75671900 -1.25397100 -0.00001700
H 2.92875700 0.26771500 -0.88152200
H 2.92875300 0.26768400 0.88154200
2_2p
E(B3LYP/6-311+G**) = -592.375674667
C -0.89196100 1.20943500 0.00179400
C 0.44970700 1.26833000 0.00723400
C 1.12323200 -0.02294400 0.00224300
C 0.11107600 -1.16418900 0.01421700
S -1.58327900 -0.41002400 -0.00784800
H 0.20228200 -1.77426700 0.91348800
H -1.56948600 2.05256300 0.00044000
H 1.00069900 2.20058000 0.01033500
C 2.45138300 -0.21282900 -0.01029200
H 2.88931800 -1.20409900 -0.01618800
H 3.13544200 0.62764500 -0.01470900
H 0.21360000 -1.80886400 -0.85898500
2_3s
E(B3LYP/6-311+G**) = -593.604666247
C -1.08594100 1.16036900 -0.21527400
C 0.39210700 1.26886000 0.19048400
C 1.01937000 -0.09782000 -0.00940700
C 0.12588300 -1.09051800 -0.04790100
S -1.57849300 -0.60371000 0.06969100
H 0.35084900 -2.14709800 -0.11694900
H -1.74247700 1.81082700 0.36099300
H 0.48828100 1.55668600 1.24747000
C 2.50913900 -0.25812400 -0.04666000
H 2.79917200 -1.30731500 -0.13145300
H 2.96945200 0.14819800 0.86249400
H -1.21743200 1.37206700 -1.27824600
H 0.89703200 2.04420600 -0.39510300
H 2.94766800 0.28518600 -0.89172200
2_3p
E(B3LYP/6-311+G**) = -593.598300545

C -0.88463500 1.22843200 -0.28105100
C 0.47695700 1.23506800 0.42391100
C 1.12395600 -0.08987400 0.10704300
C 0.13075300 -1.21395900 0.28004100
S -1.53936400 -0.49546000 -0.10008400
H 0.11830100 -1.57570200 1.31136600
H -1.59832500 1.91799400 0.16976800
H 0.32114600 1.31688800 1.50655400
C 2.37322900 -0.25537100 -0.32350000
H 2.76669100 -1.23583300 -0.56894400
H 3.04763600 0.58644500 -0.43731900
H 0.33839600 -2.05183700 -0.38499900
H -0.78051700 1.46484900 -1.34120600
H 1.09494400 2.07878900 0.10744400
3_1s
E(B3LYP/6-311+G**) = -522.918362193
C -2.08975400 0.23967900 0.00003600
C -2.31250700 -1.08089500 0.00006200
O -1.18803700 -1.89411300 -0.00007700
C -0.07073800 -1.06778900 -0.00002700
C -0.60965000 0.44906500 -0.00010300
B 0.11307200 -0.15877100 1.48551600
B 1.36265700 -1.26327500 0.90163000
B 1.36257500 -1.26340800 -0.90128900
B 0.11305400 -0.15893000 -1.48549300
B 1.75506600 0.39879900 -1.44717500
B 1.96321000 1.42131400 -0.00006800
B 0.42755800 1.47403000 0.89775200
B 1.75508700 0.39893600 1.44716100
B 2.54308900 -0.28313300 0.00000500
B 0.42752000 1.47395800 -0.89789200
C -3.09019100 1.34787500 0.00000700
H 2.69949700 2.34509900 -0.00016700
H -0.64602600 -0.42412200 2.34301800
H -0.64592800 -0.42445800 -2.34304200
H 2.31666400 0.59149300 -2.46854600
H -0.03883400 2.38782900 -1.48003000
H 1.53236000 -2.27746500 -1.47716200
H 1.53237200 -2.27741000 1.47738400
H 2.31646700 0.59179000 2.46862500
H -0.03906700 2.38785600 1.47975200
H 3.68939500 -0.56931400 -0.00003400
H -3.24344900 -1.62610200 0.00020500
H -2.96989400 1.98498600 -0.88148900
H -4.10709700 0.95223300 0.00026500
H -2.96956900 1.98527400 0.88124700
3_1p
E(B3LYP/6-311+G**) = -522.908203623
C 2.49020100 -0.97051600 -0.00256100
O 1.29899300 -1.80437500 0.00367300
C 0.16235600 -1.03108600 0.00194000
C 0.57430300 0.53013700 -0.00030500
B -0.53423100 1.48041900 0.89298300
B -1.76772100 0.31133300 1.44955000
B -2.50525600 -0.43191800 0.00040700
B -1.76720600 0.30625200 -1.45090300
B -2.05985900 1.30776800 -0.00256200
B -0.53375700 1.47728000 -0.89780000
B -0.08449400 -0.13325000 -1.47411000
B -1.25484200 -1.32431900 -0.89645200
B -1.25506500 -1.32132600 0.90088400
B -0.08514100 -0.12811200 1.47516000
C 2.06506400 0.49560900 -0.00026400
H -2.86305800 2.17416400 -0.00434900
H 0.69287500 -0.34796400 -2.33094100
H 0.69235500 -0.33975100 2.33262700
H -2.34255300 0.46292100 2.47040300
H -0.12077600 2.41944900 1.47449200
H -1.35097300 -2.34143100 1.48263600
H -1.35074100 -2.34625000 -1.47498800
H -2.34166100 0.45421000 -2.47250000
H -0.11978600 2.41402600 -1.48260600
H -3.62727800 -0.80211700 0.00086400
C 2.87470900 1.54729200 0.00088300
H 3.06493800 -1.22361000 -0.89615800
H 2.48612200 2.55786500 0.00225400
H 3.95214800 1.42896800 0.00062800
H 3.07451000 -1.22473700 0.88431100
3_2s
E(B3LYP/6-311+G**) = -269.416415194
O -1.45067600 -0.71351300 -0.00002700
C -1.47365200 0.64723000 -0.00003800
C -0.20957700 1.14217200 0.00002300
C 0.67796400 0.00723000 0.00008000
C -0.13503500 -1.08322100 0.00004000
C 2.17605800 0.02892400 -0.00003000
H 0.05504100 -2.14362000 0.00001800
H -2.44958500 1.10263700 -0.00000900
H 0.06831600 2.18511400 0.00004800
H 2.58507100 -0.98369200 -0.00037100
H 2.56589400 0.54703800 -0.88158900
H 2.56610800 0.54661600 0.88167400
3_2p
E(B3LYP/6-311+G**) = -269.392649173
O 1.46303300 -0.63260200 0.00014000
C 1.35689600 0.72186300 0.00011400
C 0.09931000 1.19287400 -0.00021700
C -0.80975500 0.05439300 -0.00005000
C 0.10714800 -1.15929400 -0.00011000
C -2.14929300 0.03630900 0.00007600
H -0.00594600 -1.78108100 -0.89096300
H 2.29943900 1.25041200 -0.00004600
H -0.18721700 2.23304500 0.00032900
H -2.70798300 -0.89247000 0.00042900
H -2.72209900 0.95572500 -0.00000200
H -0.00629400 -1.78168700 0.89026200
3_3s
E(B3LYP/6-311+G**) = -270.619956064
C 1.55556700 -0.56777200 -0.10197100
C 0.15496000 -1.18676900 0.08852400

C -0.73850900 0.03756600 -0.00885400
C 0.04747900 1.11417400 0.00641100
O 1.39573400 0.86748500 0.06541400
H -0.21814100 2.16235600 -0.00230900
H 2.29769200 -0.90497600 0.62153600
H 0.04217600 -1.67988900 1.06308000
C -2.23098900 -0.01825400 -0.03369300
H -2.66665600 0.98316300 -0.06836300
H -2.62595500 -0.52516300 0.85536200
H 1.93816800 -0.73876200 -1.11280300
H -0.06497600 -1.93635000 -0.67817100
H -2.59923200 -0.57393200 -0.90414800
3_3p
E(B3LYP/6-311+G**) = -270.610946491
C 1.43258800 -0.60625700 -0.23520100
C 0.08056100 -1.21661100 0.15565800
C -0.85297900 -0.02970000 0.02165700
C 0.03269100 1.20462600 -0.03821300
O 1.36224100 0.74784900 0.21543500
H -0.21620800 1.95480300 0.71538000
H 2.29324100 -1.07137300 0.24586700
H 0.11055300 -1.55578400 1.19663600
C -2.18027300 -0.04815100 -0.05204700
H -2.75807500 0.86590600 -0.14375000
H -2.73968400 -0.97671800 -0.02203800
H -0.02527200 1.67537600 -1.03101800
H 1.57606000 -0.63707200 -1.32540200
H -0.21406900 -2.06137600 -0.47028000
4_1s
E(B3LYP/6-311+G**) = -473.161018056
C -2.52037000 1.11452100 -0.00006100
C -2.13232500 -0.18591800 -0.00011200
C -2.99669800 -1.40050400 0.00003500
B -1.32293100 2.07780800 0.00001300
C -0.01747000 1.17899600 0.00020800
B 0.10203300 0.24750200 1.45922200
B 1.44499100 1.25728000 0.88494800
B 1.44467000 1.25727400 -0.88488400
B 0.10173600 0.24746600 -1.45898200
B 0.34299700 -1.42292300 -0.88834000
B 1.88550000 -1.45615600 -0.00011500
B 1.71764200 -0.41711200 -1.45166200
B 2.56537900 0.19225500 -0.00015200
B 1.71785000 -0.41723200 1.45154500
B 0.34307200 -1.42278400 0.88827900
C -0.62854400 -0.34260900 -0.00000700
H 2.56219700 -2.42500100 -0.00021300
H -0.62185200 0.52408300 -2.34428500
H -0.62164600 0.52382400 2.34455400
H 2.26242800 -0.64457400 2.47525900
H -0.18289200 -2.29551800 1.48323700
H 1.68321000 2.24654100 1.48115900
H 1.68291400 2.24645600 -1.48122500
H 2.26214000 -0.64427300 -2.47546000
H -0.18306700 -2.29561000 -1.48326900
H 3.72887000 0.39895300 -0.00031600
H -3.57345000 1.37363000 -0.00025600
H -2.78627000 -2.02213900 -0.87660500
H -2.78675500 -2.02091400 0.87782500
H -4.05286800 -1.13106000 0.00008700
H -1.31520600 3.26181800 -0.00024200
4_1p
E(B3LYP/6-311+G**) = -473.152087287
C -2.63900600 0.99101500 0.00143800
B -1.41592200 1.97221600 -0.00219800
C -0.09293400 1.13843800 -0.00093600
C -0.61271800 -0.45007200 0.00117100
B 0.44463900 -1.43732800 0.89093900
B 1.73673500 -0.33217000 1.45042300
B 2.54412000 0.33421600 -0.00089000
B 1.73583200 -0.33514600 -1.45030300
B 1.97911700 -1.36097300 0.00098000
B 0.44399600 -1.43910100 -0.88756900
B 0.06480800 0.21232600 -1.44391300
B 1.35669700 1.31195100 -0.88791800
B 1.35716600 1.31392600 0.88472500
B 0.06584400 0.21533600 1.44407200
C -2.11704600 -0.44408000 0.00055800
H 2.72655800 -2.27652400 0.00153200
H -0.67374300 0.44539700 -2.33277000
H -0.67215200 0.45028600 2.33288100
H 2.28896200 -0.51400500 2.47906400
H -0.01765400 -2.34051900 1.49246200
H 1.52046300 2.31700300 1.48371500
H 1.51954300 2.31370900 -1.48923900
H 2.28734500 -0.51909000 -2.47895200
H -0.01872300 -2.34336900 -1.48714900
H 3.69042100 0.62134500 -0.00151300
C -2.84923300 -1.55391900 -0.00099700
H -3.28857200 1.18387000 -0.86296600
H -2.39561000 -2.53764900 -0.00196100
H -3.93213600 -1.50644500 -0.00207100
H -1.45533900 3.15616400 -0.00518300
H -3.27889900 1.18526700 0.87300100
4_2s
E(B3LYP/6-311+G**) = -219.600500759
C 1.58720700 -0.74427000 -0.00000300
C 0.30020500 -1.11809400 0.00000400
C -0.63943100 0.08596800 -0.00000100
C 0.06453400 1.23697900 0.00000000
B 1.57950700 0.84555400 0.00000600
H -0.40515900 2.21333900 -0.00000500
H 2.52339400 1.57038400 -0.00001200
H 2.41503600 -1.44257600 -0.00001100
H -0.08311500 -2.13541600 0.00000200
C -2.11796100 -0.10969600 -0.00000100
H -2.65434300 0.84024000 -0.00001000
H -2.43033700 -0.68954200 -0.87749400
H -2.43034000 -0.68952600 0.87750100
4_2p

E(B3LYP/6-311+G**) = -219.621722153
B -1.57323500 0.72277000 0.00179900
C -1.46816600 -0.81431800 -0.00001600
C -0.14838100 -1.15112100 -0.00062400
C 0.75511100 -0.00254200 -0.00026300
C -0.08729300 1.26445100 -0.00108600
C 2.09262200 -0.09424400 0.00101000
H 0.12726100 1.89090000 -0.87691000
H -2.25149600 -1.56451300 -0.00039800
H 0.24031800 -2.16727300 -0.00142100
H 2.72412500 0.78734900 0.00116200
H 2.59289600 -1.05727700 0.00188500
H 0.13234400 1.89735200 0.86843900
H -2.56263700 1.38625500 0.00411800
4_3s
E(B3LYP/6-311+G**) = -220.854333536
C 1.65354700 -0.61403200 0.00010000
C 0.19783000 -1.13876000 -0.00005000
C -0.68910100 0.08550400 -0.00005100
C 0.00164700 1.25650900 0.00002300
B 1.50296700 0.95842800 0.00005500
H -0.51416200 2.21246400 0.00006900
H 2.22087900 -0.96848800 0.86980800
H -0.02640700 -1.76547100 0.87275100
C -2.17845600 -0.07126700 0.00000500
H -2.69060600 0.89228000 -0.00052300
H -2.50660200 -0.63987900 0.87781000
H 2.22076600 -0.96802000 -0.86990700
H -0.02611400 -1.76532200 -0.87303200
H 2.40130500 1.74347500 -0.00032400
H -2.50669800 -0.64090700 -0.87709000
4_3p
E(B3LYP/6-311+G**) = -220.835735367
C 1.49981400 -0.68202800 -0.23880200
C 0.13725700 -1.16078500 0.32955100
C -0.81582400 -0.00132600 0.09609300
C -0.02603500 1.28906700 0.22043700
B 1.43951900 0.88638900 -0.17435500
H 0.06676500 1.55809300 1.29257600
H 2.37606800 -1.16171900 0.20657600
H 0.23862500 -1.33037000 1.41014800
C -2.09413700 -0.10774100 -0.26393900
H -2.70903500 0.76789100 -0.44459200
H -2.56982100 -1.07495300 -0.38972600
H -0.48065700 2.15905600 -0.25927900
H 1.55847200 -0.91498800 -1.31688400
H -0.22744700 -2.09431000 -0.10515600
H 2.34298900 1.63623300 -0.38192700
5_1s
E(B3LYP/6-311+G**) = -541.168813195
B -2.67656300 -0.36130500 0.00000200
B -1.89251800 0.33283600 1.44466400
B -0.63756600 1.46621900 0.89203100
C 0.46393600 0.51588300 -0.00000000
C 1.94562800 0.69238000 -0.00000100
C 2.73060100 -0.40460500 0.00000800
C 2.20906700 -1.76117500 -0.00000800
N 0.97315800 -2.10994200 -0.00000600
C -0.01545800 -1.11028600 -0.00000300
B -1.46129000 -1.30137900 -0.89136600
B -1.89251800 0.33284000 -1.44466100
B -0.20949100 -0.16868100 -1.44859800
B -0.63756300 1.46621800 -0.89202000
B -2.16620100 1.35022300 0.00000500
B -0.20948900 -0.16868500 1.44859000
B -1.46129100 -1.30138200 0.89136000
H -0.21611300 2.38985600 -1.49247000
H -3.81155700 -0.69005000 -0.00000200
H -2.93330600 2.24901400 -0.00000300
H -2.44421300 0.50056800 -2.47601500
H -1.58981200 -2.31092800 1.48475700
H -1.58981100 -2.31092700 -1.48475900
H -2.44420700 0.50055300 2.47602300
H -0.21611000 2.38985600 1.49247900
H 0.54000500 -0.39045100 -2.32792900
H 0.54000200 -0.39045800 2.32792400
C 2.49304000 2.08633200 -0.00000500
H 3.80838600 -0.28831200 0.00002200
H 2.93193000 -2.57525700 0.00004800
H 3.58323300 2.07284400 -0.00000600
H 2.14551800 2.63878900 -0.87766300
H 2.14552100 2.63879300 0.87765300
5_1p
E(B3LYP/6-311+G**) = -541.152560470
C -0.05593300 -1.03956600 -0.13698900
C -0.41356600 0.59681100 0.03584900
C -1.87972400 0.91196400 0.02547300
C -2.81214200 -0.24665300 0.31792500
C -2.31386700 -1.60977900 -0.08861600
N -1.11158900 -1.97327300 -0.28138600
B 0.78083000 1.54360300 -0.72367500
B 0.24660400 0.01807900 -1.46755900
B 1.39677100 -1.25807000 -1.01365300
B 1.33998900 -1.44996900 0.75576000
B 0.16669800 -0.28122600 1.40814500
B 0.73672500 1.36225900 1.04529400
B 2.27569300 1.21441600 0.17637000
B 1.88171400 0.07589900 1.50030300
B 2.65262100 -0.51877700 0.00041900
B 1.96478000 0.38527900 -1.37813300
C -2.33517900 2.14055400 -0.20919600
H 0.34963600 2.24257500 1.72768400
H 3.75761900 -0.93728800 -0.01110000
H 3.10782000 2.04619200 0.28604400
H 2.42089900 0.08232400 2.55163800
H 1.46069200 -2.20933100 -1.70520900
H 1.37223100 -2.52475300 1.23681900
H 2.56074800 0.62163000 -2.37053400
H 0.44603000 2.55145000 -1.23552200
H -0.61463800 -0.54805700 2.24725700

H -0.49475400 -0.05298800 -2.37797400
H -3.78324700 -0.06948800 -0.15064400
H -3.40006800 2.34399000 -0.21670600
H -1.67056800 2.97411700 -0.39283600
H -3.00886600 -0.29927200 1.39911300
H -3.07208000 -2.38564500 -0.20134700
5_2s
E(B3LYP/6-311+G**) = -287.680472744
C 1.22069500 1.13762200 0.00226000
C -0.17100700 1.18950400 -0.00824100
C -0.90410400 -0.00000400 -0.01118000
C -0.17100000 -1.18950800 -0.00824100
C 1.22070100 -1.13761900 0.00226000
N 1.92231600 0.00000400 0.00813800
C -2.41092000 0.00000100 0.00748400
H -0.67510600 -2.15017400 -0.01698300
H 1.80051100 -2.05671900 0.00327100
H 1.80049900 2.05672700 0.00327200
H -0.67511900 2.15016700 -0.01698200
H -2.81554400 0.88591700 -0.48668400
H -2.81555800 -0.88579700 -0.48689100
H -2.78209500 -0.00012400 1.03798500
5_2p
E(B3LYP/6-311+G**) = -287.627749994
C 0.24539800 1.24570900 -0.11599300
C 1.02631400 0.01818600 -0.03101600
C 0.21770000 -1.24647700 -0.24144400
C -1.23887700 -1.07763200 0.11832400
N -1.85308400 0.04042000 0.17859200
C -1.09977200 1.21161500 -0.04961300
H 0.24677300 -1.51598300 -1.31018400
H -1.81936500 -1.98037000 0.31242500
H -1.69134600 2.11793700 -0.10313200
H 0.76337700 2.19644700 -0.18533000
C 2.35040800 0.01111700 0.18564200
H 2.90922800 0.93659200 0.26957600
H 2.90941900 -0.91314200 0.27983000
H 0.64648200 -2.09952100 0.29127100
5_3s
E(B3LYP/6-311+G**) = -288.859118412
C 0.18722400 1.19337200 0.28148400
C 0.95304900 -0.08148600 0.00795100
C 0.24466600 -1.21986900 -0.07624100
C -1.21305900 -1.19109800 0.10329300
N -1.94396300 -0.14392200 0.05141200
C -1.24123800 1.10497500 -0.26564000
H -1.71784100 -2.13962100 0.29483600
H -1.22181900 1.18957400 -1.36252100
H 0.69896600 2.05681300 -0.15578200
C 2.44740500 -0.01717100 -0.07093600
H 2.86134700 0.42930900 0.84143400
H 2.89196900 -1.00538900 -0.20378800
H 0.73077400 -2.17978600 -0.22033200
H -1.84489900 1.93841900 0.10009400
H 0.17434800 1.36388300 1.36877800
H 2.76661400 0.62191100 -0.90206200
5_3p
E(B3LYP/6-311+G**) = -288.850082325
C -0.24709000 -1.17480500 0.52622500
C -1.05453000 0.02340600 0.10899100
C -0.29061200 1.31258900 0.27043100
C 1.18541100 1.15040000 -0.04318900
N 1.81352600 0.07323100 -0.27309500
C 1.08998600 -1.19790800 -0.23925500
H -0.70585700 2.10747100 -0.35770400
H 1.76612700 2.07624100 -0.07548300
H 0.92611400 -1.50278300 -1.27987400
H -0.79687700 -2.10473000 0.35987100
C -2.28702000 -0.04210300 -0.39408400
H -2.80449500 -0.98998200 -0.49862900
H -2.82166800 0.84679000 -0.71275200
H -0.36868900 1.67726200 1.30619900
H 1.76246200 -1.94357400 0.19473300
H -0.02868100 -1.10879400 1.60059000
6_1s
E(B3LYP/6-311+G**) = -525.128166119
B 2.69090300 -0.38869200 -0.00022300
B 1.91586500 0.31785100 -1.44553600
B 0.66142700 1.44819500 -0.89239000
C -0.45638000 0.49173200 0.00062200
C -1.93476400 0.72584000 0.00024500
C -2.78062900 -0.32538200 0.00021700
C -2.36370700 -1.71416300 -0.00000800
C -1.07529300 -2.09065800 -0.00016300
C 0.01706100 -1.09546500 -0.00010000
B 1.46233700 -1.30946700 0.89146700
B 1.91625100 0.31763400 1.44519800
B 0.22921900 -0.17062800 1.46189100
B 0.66166900 1.44822700 0.89258800
B 2.19076200 1.32880500 -0.00015200
B 0.22887400 -0.17061500 -1.46141800
B 1.46219100 -1.30950900 -0.89175400
H 0.24665000 2.37979300 1.48413200
H 3.82224600 -0.73059400 -0.00025400
H 2.96040400 2.22579400 -0.00021300
H 2.47180600 0.48056200 2.47542200
H 1.58547000 -2.32599200 -1.47887200
H 1.58558000 -2.32581700 1.47883800
H 2.47095500 0.48097600 -2.47597200
H 0.24574300 2.37962300 -1.48368500
H -0.51607900 -0.39082600 2.34466800
H -0.51658800 -0.39041500 -2.34416700
C -2.41840800 2.14731200 -0.00023000
H -3.84649900 -0.12489800 0.00004100
H -0.77833500 -3.13173800 -0.00024800
H -3.13597900 -2.47491200 -0.00016700
H -3.50865000 2.17682700 0.00196700
H -2.05387900 2.68968900 0.87660200
H -2.05761300 2.68761900 -0.87993800
I_1p

E(B3LYP/6-311+G**) = -525.111173866
B -2.66540300 -0.54495800 -0.01085400
B -2.00032700 0.38762400 1.36210800
B -0.81610100 1.53940000 0.70449900
C 0.40089200 0.57699600 -0.02998100
C 1.86445900 0.93102200 -0.02077400
C 2.84698400 -0.16758400 -0.38851500
C 2.46714100 -1.54888700 0.07491100
C 1.21628300 -1.93766300 0.31025400
C 0.05077900 -1.02650900 0.15267600
B -1.33782400 -1.46552300 -0.74624700
B -1.89218000 0.04417600 -1.51191300
B -0.17490100 -0.30378200 -1.41099500
B -0.75073700 1.33384700 -1.06091100
B -2.30018600 1.19126500 -0.20525100
B -0.28178200 0.03870200 1.48058200
B -1.40524500 -1.25257300 1.02013700
H -0.36186200 2.21231200 -1.74427500
H -3.76645500 -0.97434300 -0.00101400
H -3.13526400 2.01789700 -0.33231800
H -2.42350600 0.03539500 -2.56735900
H -1.47193500 -2.20007500 1.72060000
H -1.36162400 -2.54992800 -1.21018600
H -2.60935700 0.62983600 2.34530000
H -0.49689500 2.56013100 1.19925400
H 0.61056200 -0.57204900 -2.24419900
H 0.44555200 -0.01389600 2.40257400
C 2.27500600 2.16440800 0.26892200
H 3.83046800 0.09856600 0.00549500
H 3.33184000 2.40593300 0.27172600
H 1.58542800 2.96447100 0.50171200
H 0.98335600 -2.94592600 0.63061300
H 2.96108700 -0.19383000 -1.48225000
H 3.27276200 -2.26608500 0.19360000
I_2s
E(B3LYP/6-311+G**) = -271.638813491
C 1.19958500 1.20343200 0.00206400
C -0.19416000 1.20055900 -0.00906900
C -0.91306600 0.00002900 -0.01156900
C -0.19419200 -1.20054100 -0.00907200
C 1.19953700 -1.20345900 0.00206600
C 1.90269100 -0.00001800 0.00862100
C -2.42309800 0.00001100 0.00950200
H -0.73190100 -2.14360000 -0.01827600
H 1.73592600 -2.14612400 0.00175400
H 2.98682900 -0.00004100 0.01433800
H 1.73599600 2.14608500 0.00175200
H -0.73183300 2.14363700 -0.01827500
H -2.82901100 0.88504800 -0.48613100
H -2.82902100 -0.88375200 -0.48841000
H -2.80077500 -0.00133200 1.03798800
I_2p
E(B3LYP/6-311+G**) = -271.584869882
C -0.27655700 -1.26177400 -0.11004000
C -1.03203900 -0.01481400 -0.02744600
C -0.24014000 1.27472900 -0.19658900
C 1.23392700 1.14652200 0.08782800
C 1.83758500 -0.04919400 0.12934200
C 1.07055400 -1.27969300 -0.04056200
H -0.34584800 1.61070500 -1.24079500
H 1.80499900 2.06158800 0.20708300
H 2.90713100 -0.12182800 0.29461100
H 1.60364200 -2.22383100 -0.06939600
H -0.83667000 -2.18940500 -0.17271600
C -2.36121400 -0.01249700 0.16930100
H -2.92138100 -0.93910500 0.22941300
H -2.92294900 0.90934900 0.27277200
H -0.68161500 2.07285600 0.40802600
I_3s
E(B3LYP/6-311+G**) = -272.814159828
C 0.20410100 1.19938700 0.28839000
C 0.96056100 -0.08482100 0.00814800
C 0.27238000 -1.23812200 -0.05923100
C -1.18372000 -1.26943400 0.09239100
C -1.91081800 -0.14491800 0.02159800
C -1.23212300 1.17347100 -0.25714900
H -1.66654900 -2.22964100 0.24358300
H -2.99275200 -0.17457600 0.09897400
H -1.21492400 1.33283600 -1.34689700
H 0.74459200 2.05826400 -0.12175300
C 2.45620900 -0.00802800 -0.07983500
H 2.87729600 0.44311300 0.82783300
H 2.90581700 -0.99488600 -0.20948300
H 0.79722500 -2.17816900 -0.20249700
H -1.80610400 2.00523800 0.16111500
H 0.18347300 1.34675700 1.37998600
H 2.77238600 0.62585500 -0.91673600
I_3p
E(B3LYP/6-311+G**) = -272.806416685
C -0.25690000 -1.18537100 0.53868200
C -1.05225100 0.01818200 0.10007400
C -0.33199600 1.33409400 0.29130100
C 1.13966900 1.23741200 -0.03683500
C 1.76921000 0.08534700 -0.27291900
C 1.08892200 -1.25805900 -0.21087800
H -0.80135700 2.11389100 -0.31764100
H 1.69199500 2.17223600 -0.08250900
H 2.82610700 0.09701200 -0.52597700
H 0.92753200 -1.63482800 -1.22966700
H -0.83074900 -2.10373600 0.39206100
C -2.27124700 -0.06742100 -0.43356300
H -2.76982100 -1.02351600 -0.55599600
H -2.81369200 0.81359200 -0.76125700
H -0.44232100 1.66528900 1.33570600
H 1.74723500 -1.98579400 0.27693200
H -0.04737200 -1.09924400 1.61317800
